# The Critical Role of MMP13 in Regulating Tooth Development and Reactionary Dentinogenesis Repair Through the Wnt Signaling Pathway

**DOI:** 10.3389/fcell.2022.883266

**Published:** 2022-04-21

**Authors:** Henry F. Duncan, Yoshifumi Kobayashi, Yukako Yamauchi, Angela Quispe-Salcedo, Zhi Chao Feng, Jia Huang, Nicola C. Partridge, Teruyo Nakatani, Jeanine D’Armiento, Emi Shimizu

**Affiliations:** ^1^ Division of Restorative Dentistry & Periodontology, Dublin Dental University Hospital, Trinity College Dublin, Dublin, Ireland; ^2^ Department of Oral Biology, Rutgers School of Dental Medicine, Newark, NJ, United States; ^3^ School of Stomatology, Universidad Cientifica del Sur, Lima, Peru; ^4^ Department of Molecular Pathobiology, New York University Dentistry, New York, NY, United States; ^5^ Department of Physiology and Cellular Biophysics, Columbia University Medical Centre, New York, NY, United States

**Keywords:** collagenase, dentinogenesis, histone deacetylase, matrix metalloproteinase, odontoblast, Wnt signaling

## Abstract

Matrix-metalloproteinase-13 (MMP13) is important for bone formation and remodeling; however, its role in tooth development remains unknown. To investigate this, MMP13-knockout (*Mmp13* ^*−/−*^) mice were used to analyze phenotypic changes in the dentin–pulp complex, mineralization-associated marker-expression, and mechanistic interactions. Immunohistochemistry demonstrated high MMP13-expression in pulp-tissue, ameloblasts, odontoblasts, and dentin in developing WT-molars, which reduced in adults, with human-DPC cultures demonstrating a >2000-fold increase in *Mmp13*-expression during mineralization. Morphologically, *Mmp13* ^*−/−*^ molars displayed critical alterations in the dentin-phenotype, affecting dentin-tubule regularity, the odontoblast-palisade and predentin-definition with significantly reduced dentin volume (∼30% incisor; 13% molar), and enamel and dentin mineral-density. Reactionary-tertiary-dentin in response to injury was reduced at *Mmp13* ^*−/−*^ molar cusp-tips but with significantly more dystrophic pulpal mineralization in MMP13-null samples. Odontoblast differentiation-markers, nestin and DSP, reduced in expression after MMP13-loss *in vivo*, with reduced calcium deposition in MMP13-null DPC cultures. RNA-sequencing analysis of WT and *Mmp13* ^*−/−*^ pulp highlighted 5,020 transcripts to have significantly >2.0-fold change, with pathway-analysis indicating downregulation of the Wnt-signaling pathway, supported by reduced *in vivo* expression of the Wnt-responsive gene Axin2. Mmp13 interaction with Axin2 could be partly responsible for the loss of odontoblastic activity and alteration to the tooth phenotype and volume which is evident in this study. Overall, our novel findings indicate MMP13 as critical for tooth development and mineralization processes, highlighting mechanistic interaction with the Wnt-signaling pathway.

## Introduction

Matrix metalloproteinase-13 (MMP13) or collagenase-3 is important in endochondral-ossification, bone-remodeling, and dental pulp mineralization processes (Inada et al., 2004; Duncan et al., 2016). MMP13-deficient mice exhibit altered long bone formation (Stickens et al., 2004), while in histone deacetylase (HDAC)-4-deleted mice, MMP13-expression is elevated in both chondrocytes and trabecular bone, producing an altered-phenotype attributed to an overexpression-induced bone-remodeling disorder (Nakatani et al., 2016). Within dental pulp cell (DPC) cultures, MMP13-expression is high in pulp tissue (Palosaari et al., 2003) and further increased during pulp mineralization (Suri et al., 2008), with pharmacological MMP13-inhibition altering DPC mineralization *in vitro* (Duncan et al., 2016).

Previous research from our group has highlighted the significant interplay between class II HDAC and MMP13 in mineralizing tissue, with MMP13-expression suppressed by HDAC4 in bone (Shimizu et al., 2010). Conversely, an increase in *Mmp13* mRNA and protein expression mediated the effect of HDAC4-deletion on the skeleton (Nakatani et al., 2016). The application of an HDAC-inhibitor (HDACi), suberoylanilide-hydroxamic-acid, to DPC cultures stimulated the upregulation of both *MMP13* gene and protein expression during mineralization *in vitro*, while MMP13-inhibition further altered the expression of mineralization-associated markers including bone morphogenetic protein 4 (*Bmp4*), osteopontin (*Opn*), and the protease *Mmp9* expression (Duncan et al., 2016). Previous studies investigating the deletion of MMPs on tooth development have highlighted the altered dentin structure with *Mmp9* (Yuan et al., 2017) and enamel structure with *Mmp20* (Bartlett et al., 2006); however, the effect of *Mmp13*-deletion on dentinogenesis and tooth development *in vivo* remains unknown. Although MMP13 has been linked to mineralization processes in tooth (Duncan et al., 2016) from a mechanistic perspective, the regulators of MMP13 activity remain to be elucidated. The activity of MMP20 has been linked to *Wnt* signaling (Shin et al., 2018) and JNK/c-jun (Zhang et al., 2007) in enamel development, while dentin sialoprotein (DSP) was identified as a novel substrate of MMP9 in developing teeth (Yuan et al., 2017).

In this study, we show that MMP13 is highly expressed in dental tissues during development and hypothesize that its expression regulates the ordered formation of dentin *in vivo* and repair after cuspal wear. Furthermore, we demonstrate that MMP13-deletion will affect DPC proliferation, tooth development, and the expression of ameloblastic, odontoblastic, and mineralization-associated gene and protein markers including other proteases and HDACs while highlighting its regulation through Wnt signaling and the odontoblast-responsive Axin2 expression.

## Materials and Methods

### Animals

Homozygous MMP13-deficient mice (*Mmp13* ^*−/−*^) on a C57BL/6 background were gifted by Dr. D’Armiento. Male WT and *Mmp-13* ^*−/−*^ mice were characterized and housed as described previously (Nakatani et al., 2016). Experimental (*Mmp13* ^*−/−*^) and control (WT) mice were sacrificed by CO_2_ narcosis at a range of time-points (1 day postnatal to 3 months). Animal weights were recorded before experimentation and subsequently. All experiments followed protocols approved by the New York University Institutional Animal Care and Use Committee (IACUC). All the animal work was complied with the Animal Research: Reporting *In Vivo* Experiments (ARRIVE) guidelines.

### Dental Pulp Cell Culture and the Characterization

Mouse dental pulp cells (DPCs) were isolated from molar and incisor teeth of *Mmp13* ^*−/−*^, and WT was homogenized with TRIzol reagent (Thermo Fisher Scientific, Waltham, MA, United States) and 6 mm zirconium oxide (Thomas Scientific, Swedesboro, NJ, United States) using the bead-based homogenizer (BeadBug, Thomas Scientific) and incubated in PBS containing 3 mg/ml collagenase A (Sigma-Aldrich, St. Louis, MO, United States) and 2.5 mg/ml trypsin (Sigma Aldrich) for 30 min at 37°C. Human dental pulp cells (HDPCs) were purchased from Lonza (Morristown, NJ, United States) and were recently authenticated and verified to be contamination-free. Mouse DPCs and HDPCs were cultured in alpha-MEM with 20% fetal bovine serum, 2 mM L-glutamine, and 100 U/ml penicillin–streptomycin (Life Technologies, Grand Island, NY, United States) at 37°C in 5% CO_2_. The colony-forming unit fibroblast (CFU-F) assay followed a modification of a previous protocol (Chou et al., 2009). A total of 2.0 × 10^3^ cells were cultured in a six-well plate for 14 days, followed by Giemsa staining, colony counting, and analysis. For cell proliferation, 5.0 × 10^4^ cells were cultured in serum-free media in a 12-well plate. After 1-day, the media was changed to include serum (day-0), and cell numbers were counted using a hemocytometer daily until day-5. The Vybrant succinyl dehydrogenase (3-(4,5-dimethylthiazol-2-yl)-2,5-diphenyl tetrazolium bromide (MTT) Cell Proliferation Assay Kit (Molecular Probes, Eugene, OR, United States) was used according to manufacturer’s protocol. For DPC differentiation, mouse or human DPCs were cultured with differentiation media containing alpha-MEM (as aforementioned) plus 2 mM L-glutamine, 10 nM dexamethasone, 50 μg/ml L-ascorbic acid, and 10 mM beta-glycerophosphate (Sigma-Aldrich, St. Louis, MO, United States) for up to 21 days. Beta-glycerophosphate was only used for the last 3–4 days of culture. After differentiation, the cells were fixed by 4% paraformaldehyde, stained with Alizarin red solution, washed three times with distilled water, and microscopically analyzed.

### Histological Analysis

Mouse-mandibles were dissected for micro-computed tomography (µCT) assessment, while the remainder of the head was fixed in 10% buffered formalin at 4°C for 24-h, prior to decalcification in 10% EDTA. The fixed-tissue was dehydrated through ascending concentrations of ethanol, paraffin-embedded, and serially sectioned (5 μm). Thereafter, the comparative sections were deparaffinized, hydrated, and stained with hematoxylin and eosin (H&E) (Sigma-Aldrich) prior to morphological analysis at 10-day, 3-week, 6-week, and 3-month using a Zeiss Axio (Carl Zeiss, Jena, Thuringia, Germany) light microscope.

### Immunohistochemical Analysis

To detect the expression and distribution of the mineralization-related markers; amelogenin, Axin2, nestin, dentin sialoprotein (DSP), proteases (MMP8, MMP9, and MMP13), and class-II HDAC (4 and 5), IHC was carried out in similar sections using the Envision + horseradish peroxidase (HRP) staining system (Dako, Agilent, Cork, Ireland) according to the manufacturer’s instructions. Briefly, deparaffinized and hydrated sections were rinsed in phosphate-buffered-saline (PBS) prior to endogenous peroxidase activity being blocked with a 0.3% hydrogen peroxide solution for 20 min (Dual Endogenous Enzyme Block, Dako). The sections were rinsed and blocked using a 1% bovine serum albumin (BSA) solution with 2% goat serum (Santa Cruz Biotechnology, Heildelberg, Germany), and 0.05% Tween-20 (Bio-Rad, Hertfordshire, United Kingdom) for one-hour at room temperature. The sections were incubated with primary antibody in a TBS solution with 1% BSA overnight at 4°C in a humidifying chamber. MMP13-expression was analyzed in WT mice using anti-MMP13 [Abcam, Cambridge, United Kingdom; Cat. number ab39012; Batch number GR157514; Dilution used 1:100; (Soundia et al., 2018)] at 1, 3, 6, and 9-day post-natal. The sections from *Mmp13* ^*−/−*^ and WT molars at postnatal day 3, day 10, or 3 months were incubated with anti-amelogenin [Abcam; Cat. number ab153915; Batch number GR114097; Dilution used 1:200; (Pandya et al., 2017)], anti-Axin2 [Abcam; Cat. number ab32197; Batch number GR3363772; Dilution used 1:200; (Zhang et al., 2020)], anti-DSP [Millipore, Temecula, CA, United States; Cat. number MABT37; Lot number 2844589; Dilution used 1:200; (Baba et al., 2004)], anti-nestin [Millipore; Cat. number MAB353; Lot number 2370131; Dilution used 1:200; (Hiraga et al., 2010)], anti-MMP8 [Abcam; Cat. number ab53017; Batch number GR38794; Dilution used 1:100; (Zhu et al., 2019)] anti-MMP9 [Abcam; Cat. number ab38898; Batch number 573145; Dilution used 1:100; (Soundia et al., 2018)], anti-HDAC4 [Abcam; ab12172; Batch number GR285278; Dilution used 1:200; (Wein et al., 2016)], and anti-HDAC5 [Abcam; Cat. number ab55403; Batch number GR36969; Dilution used 1:100; (Wanek et al., 2018)] antibodies or PBS replacing primary antibodies as a negative control. The positive control was demonstrated by antibody-expression in tissues previously shown to have a high expression. After washing, the sections were placed in HRP-labeled polymer conjugated to goat anti-rabbit and anti-mouse secondary antibodies (Envision+, Dako) for 30 min, prior to the completion of staining with a 5-min incubation with 3,3′-diaminobenzidine (DAB) chromagen solution, washing in distilled water and counterstaining with hematoxylin (Sigma-Aldrich). As before, a Zeiss Axio (Carl Zeiss) light microscope was used.

### Micro-Computed Tomography

The mandibles of *Mmp13* ^*−/−*^ and WT mice were fixed in 70% ethanol and prepared for high-resolution µCT (SkyScan 1172, Bruker, Kontich, Belgium) of incisor and first molar teeth. A three-dimensional analysis was carried out to determine total volume, enamel volume, dentin volume, pulp volume, and total mineral density (TMD). The samples were scanned using a 10-MP digital detector, 10W of energy (70 kV and 142 mA), and a pixel size of 7.5microns, exposure 850 ms/frame rotation step 0.3° with ×10 frame averaging, 0.5 mm aluminum filter, and scan rotation of 180°. After scanning, the radiographs were reconstructed using NRecon software (version 1.7.3.0; Bruker). Reconstruction was conducted with NRecon using GPU acceleration. Gaussian smoothing was applied with a 2-voxel radius, ring artifact and beam hardening corrections were applied in reconstruction. Ring artefact reduction set to 7 pixels. Beam hardening correction was set to 40%. CTAn software (CTAn Micro-CT software, Bruker) was used to generate 2-D images for color density and 3-D images for CT volume.

### Quantitative Real-Time PCR

Total RNA from cultured pulp cells was isolated using the TRIzol (Thermo Fisher Scientific) method, and reverse transcribed to complementary DNA (cDNA) with TaqMan Reverse Transcription Reagents (Thermo Fisher Scientific) according to the manufacturer’s instructions. The sequences were amplified by adding complementary DNA to the PCR mixture containing each primer (listed Supplementary Table S1) and Platinum SYBR Green qPCR SuperMix uracil-DNA glycosylase (UDG) (Thermo Fisher Scientific). The reactions were pre-incubated at 50°C for 2 min for decontamination of deoxyuridine (dU)-containing DNA by UDG and then incubated at 95°C for 2 min to inactivate UDG and activate Taq. The PCR program continued 46 cycles of denaturation at 95°C for 15-sec, annealing at 60°C for 30-sec, and elongation at 72°C for 30-sec. All data were normalized using the Ct value of beta-actin gene expression from the same sample.

### RNA Preparation, RNA-Sequencing, and Data Analysis

The Illumina HiSeq 2500 system (Illumina, Inc., San Diego, CA, United States) was used to analyze the transcript profiles of pulp tissue isolated from the incisors of *Mmp13* ^*−/−*^ and WT mice at 3 months. The RNAseq analysis was performed on three independent biological samples for each genotype (*n* = 3) and run on a 2 × 150 bp configuration and single index per lane in a paired end experimental design. The pulpal tissue was homogenized [T10 basic S2-Ultra-Turrax tissue disrupter (IKA, Staufen, Germany)], and the total RNA was extracted using the TRIzol reagent (Thermo Fisher Scientific, Wilmington, DE, United States) and quantified spectrophotometrically (Nanodrop 2000, Thermo Fisher Scientific). RNA-seq was performed according to the manufacturer’s instructions, and raw data were exported to Strand-NGS ver4.0 (Strand Life Sciences, Bengaluru, India) for subsequent analysis. Genes that were differentially expressed (>2.0 fold) in the MMP13KO group relative to the WT control were identified after passing a t-test (*p* < 0.05) and post hoc test (Storey with Bootstrapping) with a corrected q-value of 0.05. Genes in the expression data sets were first “ranked” based on Log2 values from highest to lowest for both groups at both time points, prior to hierarchical clustering being used to group gene expression in each condition using the default settings in Strand-NGS. Gene Ontology (GO) was evaluated using Go-Elite (http://www.genmapp.org/go_elite) (Zambon et al., 2012), which is designed to identify a minimal non-redundant set of biological ontology terms or pathways to describe a particular set of genes or metabolites. The subsequent pathway analysis was undertaken using Pathvisio (http://www.pathvisio.org/ - version 3.3.0+) (Kutmon et al., 2015), which uses an over-representation analysis, only reporting on GO terms and pathways with a z score >2, a permutation *p* < 0.01, and three or more regulated genes for the pathway. This data was then linked and illustrated using WikiPathways (Slenter et al., 2018). RNA-sequencing data followed the Minimum Information About a Sequencing Experiment (MINSEQE) guidelines and have been submitted to the Gene Expression Omnibus (GEO), accession number: GSE178898.

### Western Blot

The analysis was carried out on cell lysates from DPCs isolated from 3-month old mouse molars (*Mmp13* ^*−/−*^) and cultured in (proliferation, 5-day culture) mineralization media for 14 days (differentiation). The protein lysates were prepared by incubating the cells for 30 min at 4°C in extraction buffer, comprising RIPA buffer, phenylmethyl sulfonyl fluoride (PMSF), and halt protease inhibitor (all from Thermo Fisher Scientific, Waltham, MA, United States), prior to centrifugation at 13,000 rpm for 10 min at 4°C. Total protein was quantified by a Bradford assay (Bio-Rad Laboratories GmbH, München, Germany) using the NanoDrop 2000 spectrophotometer (Thermo Fisher Scientific), before 50–100 µg proteins were used for sodium dodecyl sulfate–polyacrylamide gel electrophoresis (SDS-PAGE) on a 4%–15% polyacrylamide gel (Mini-PROTEAN, Bio-Rad) and the migrated proteins were transferred to the polyvinylidene difluoride (PVDF) membrane. To block non-specific protein binding, the membrane was incubated in 5% non-fat dry milk at room temperature for 1 h, prior to overnight incubation at 4°C with primary antibodies raised against HDAC4 (Cell signaling technology; Cat. number 15164; Dilution used 1.1,000), HDAC5 (Cell signaling technology; Cat. number 20458; Dilution used 1.1,000), HDAC6 (Cell signaling technology; Cat. number 7612; Dilution used 1.1,000), Axin2 (Abcam; Cat. number ab32197; Dilution used 1.1,000), phospho-β-catenin (Cell Signaling Technology; Cat. number 9561; Dilution used 1:2,000), β-catenin (Cell Signaling; Cat. number 8480; Dilution used 1:1,000) phospho-p38 (Cell Signaling Technology; Cat. number 9211; Dilution used 1:1,000), p38 (Cell Signaling Technology; Cat. Number 9212; Dilution used 1:1,000), and β-actin (Cell Signaling Technology; Cat. number 4967; Dilution used 1:1,000) in 1:1,000 solution in 2.5% skimmed milk. After washing, the membrane was incubated with 2ry antibodies at room temperature for 2 h, developed using an enhanced chemiluminescence (ECL) detection kit (Clarity Western, Bio-Rad), and then detected using the Chemidoc Touch Imaging System (Bio-Rad). The protein expression was evaluated using Fluor Chem R Hy8300 (ProteinSimple) with β-actin used as a loading control to normalize the data. In total, three independent experiments (*n* = 3) were carried out for each HDAC target and repeated in triplicate. The quantitative results were obtained using the image analyzing software ImageJ.

### Statistical Analysis

The Student’s t-test was used for quantitative analysis of µCT, CFU-F, MTT assay, and qRT-PCR data (*p* < 0.05). The results are expressed as mean ± standard deviation. The presence or absence of dystrophic pulp calcification in *Mmp13* ^*−/−*^ and WT was determined using contingency tables and a Fisher’s exact test (*p* < 0.05). Data analysis used IBM SPSS (v25, Dublin, Ireland) (*p* < 0.05). Differential expression in RNAseq experimentation was identified after passing a t-test (*p* < 0.05) and post hoc test (Storey with Bootstrapping) with a corrected *q*-value of 0.05. The number of independent experiments or animals is listed in the results and figures.

## Results

### MMP13 Is Highly Expressed During Murine Tooth Development and in Mineralizing Human Dental Pulp Cells

**FIGURE 1:**
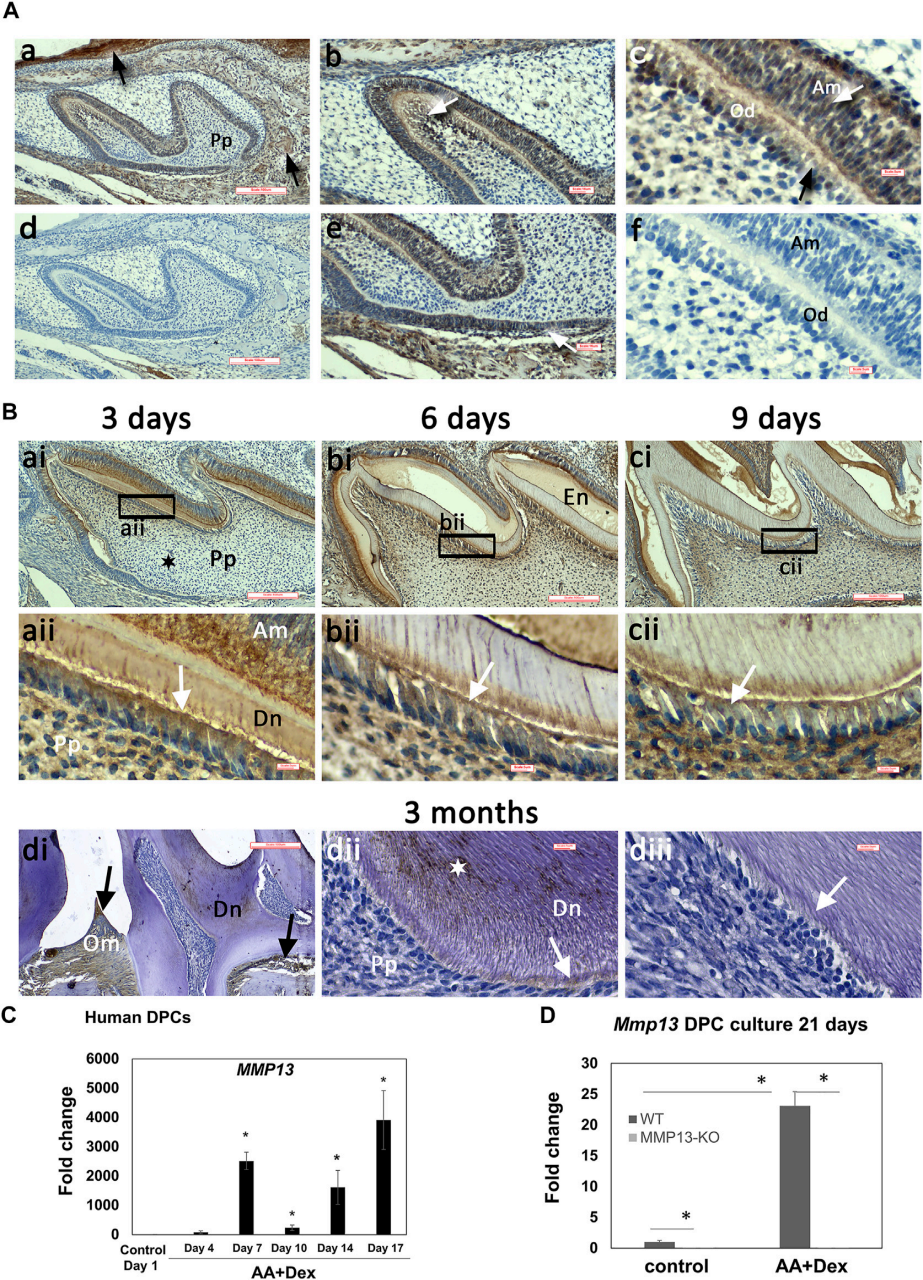
High MMP13-expression in the dentin–pulp complex of developing mouse molars and in mineralizing human-DPC cultures. IHC analysis of MMP13 in sagittal tooth sections of maxillary first molars (postnatal 1, 3, 6, 9-day, and 3-month). **(A)** Postnatal 1-d **(a, b, c, e)** and unstained controls **(d, f)**. MMP13 in oral mucosa and alveolar bone is highlighted in (**a**—black arrows), in odontoblasts highlighted in (**c**—black arrow), in sub-odontoblastic layer highlighted in (**b**- white arrow), in ameloblasts highlighted in (**c**—white arrow), and root sheath of Hertwig highlighted in (**e**—white arrow). Scale bars = **(a, d)** 100 μm (original mag. ×10), **(b, e)** 16 μm (original mag. x20), and **(c, f)** 5 µm (original mag. ×63). **(B)** Continued high MMP13 expression at postnatal 3-, 6-, and 9-day **(a–c)**. Low central pulp MMP13 expression at 3-day postnatal is highlighted **(ai)** by a black star. MMP13 expression in 3-month mouse molars is highlighted in the mineralized dentin (**dii**—white star). Positive control MMP13 expression in oral mucosa and alveolar bone (**di**—black arrows) is noted, while MMP13 expression in the odontoblast layer and predentin layer of mature molars (**dii**– white arrows; **diii**- negative control) is low compared with developing teeth (**aii–cii**—white arrows). aii–cii represents higher magnifications of the black boxes in **ai–ci**, respectively. Am, ameloblast; En, enamel; Od, odontoblasts; Om, oral mucosa; Pp, pulp. Sections from five WT mice at 1-, 3-, 6-, and 9-day and nine WT mice at 3-month. Scale bars = **(ai, bi, ci, di)** 100 μm (original mag. ×10) and **(aii, bii, cii, dii, diii)** 5 µm (original mag. ×63). **(C)** HDPCs cultured with or without ascorbic acid and dexamethasone over 17 days and RNA collected in time-course highlights significant increase in *Mmp13* expression during mineralization over control at all time-points after day 4 *p < 0.05 versus control at day-1. **(D)** Culture from WT and *Mmp13* ^*−/−*^ incisors indicates a large increase in WT *Mmp13* expression during mineralization at 21-day but negligible *Mmp13* expression in MMP13KO mice. All data are expressed as mean ± s.d., *p < 0.05 versus control.

MMP13-expression was analyzed in developing (1, 3, 6, and 9-day) and adult (3-month) WT maxillary first-molars by immunohistochemistry (IHC) and qRT-PCR. MMP13 was detectable 1-day postnatally with strong staining evident in preodontoblasts, odontoblasts, ameloblasts, and the root sheath of Hertwig, compared with the unstained control **(** Figure 1A). MMP13-expression persisted at 3, 6, and 9-day with increased detection in the odontoblast, ameloblast layer, and mineralized-dentin (Figure 1B), while exhibiting comparatively low expression in the central pulp and perivascular area at all-time points (Figure 1B). MMP13-expression in 3-month mice was evident in the mineralized-dentin than that in the unstained control (Figures 1Bdii,diii), while expression remained high at 3-month in constantly remodeling tissues such as oral mucosa and alveolar bone (Figure 1Bdi). MMP13-expression was reduced in the odontoblast and predentin of adult molars (Figure 1Bd) than that in developing teeth (Figure 1B), suggesting a role for MMP13 in odontoblast-activity and differentiation. *Mmp13* gene-expression was also significantly increased (*p* < 0.05) in mineralizing-hDPC cultures and differentiated-hDPCs compared with untreated DPC controls (Figure 1C), while MMP13-ablation eliminated *Mmp13* gene expression (Figure 1D).

MMP13 was highly-expressed in alveolar bone and oral mucosa, positively confirming tissues reported to have a high MMP13-expression (Uitto et al., 1998) (Figure 1Aa).

### MMP13-Loss Causes Abnormal Dental Phenotype and Reduces Dentin Volume and Density

**FIGURE 2:**
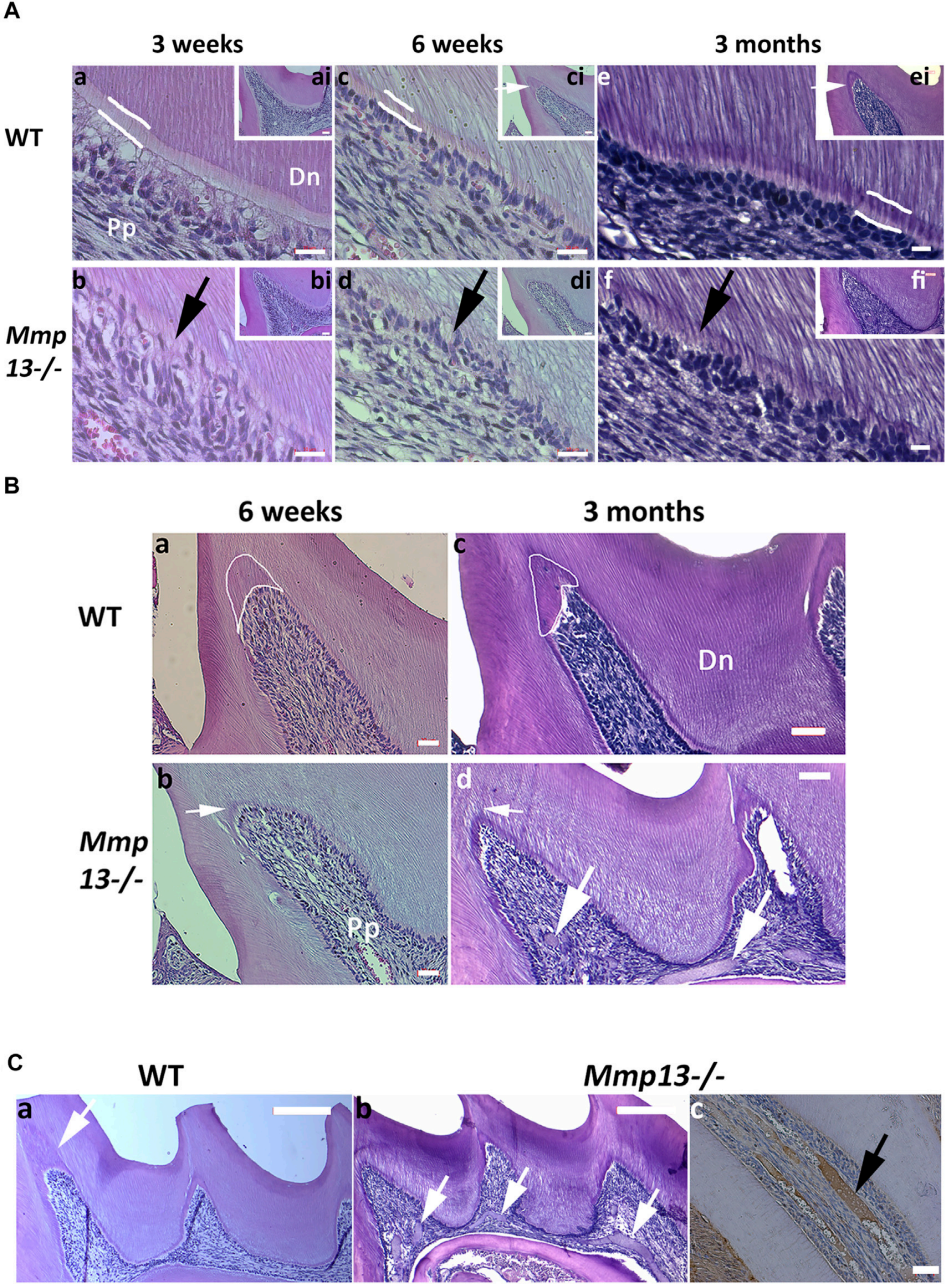
MMP13-ablation alters dentin phenotype, reducing volume and density. **(A)** Comparative histological analysis of representative sagittal sections of maxillary first molars from *Mmp13* ^*−/−*^ and WT mice at postnatal 3-week, 6-week, and 3-months. **(a–f)** H&E staining demonstrates the altered dentin structure. Analysis of WT **(a, c, e)** and *Mmp13* ^*−/−*^ **(b, d, f)** mouse molars at 3-week, 6-week, and 3-month highlights progressively altered the dental phenotype including alterations in dentin tubule regularity, disruption of odontoblast layer organization and palisade (**d–h**. black arrow), narrowing of the predentin layer (indicated sketched white lines in **a, c, e**), and reduced reactionary dentin formation (**ci, ei**. [inset] small white arrows). **a–f** represent higher magnification of distal cusp (inset; **ai–fi**). Scale bars = **(ai–fi)** 20 μm (original mag. ×20); **(a–f)** 10 μm (original mag. x63). **(B)** Reactionary dentinogenesis was analyzed in 6-week and 3-month maxillary first molar teeth of WT and *Mmp13* ^*−/−*^ mice using H&E staining of sagittal sections. Reactionary dentin is reduced after MMP13-ablation (**Bb, Bd**—white arrows) compared with WT (**Ba, Bc**—white encircled areas; **Ca**—white arrow). Multiple pulpal calcifications identified in 3-month *Mmp13* ^*−/−*^ molars (**(Cb)**—white arrows; **(Cc)**—black arrows). Positive staining for HDAC5 highlights pulpal mineral to be cemental- or bone-like rather than dentin-like in nature with no evident tubular structure (**Cc**—black arrow). Scale bars = **(Ba, Bb, cc)** 20 μm (original mag. ×20); **(Bc, Bd)** 16 μm (original mag. ×20); **(Ca, Cb)** 50 μm (original mag. ×10). Pp, pulp tissue; Dn, dentin. Sections were examined from six mice per genotype at postnatal 3-week and 6-week and nine mice per genotype at 12-week.

**FIGURE 3:**
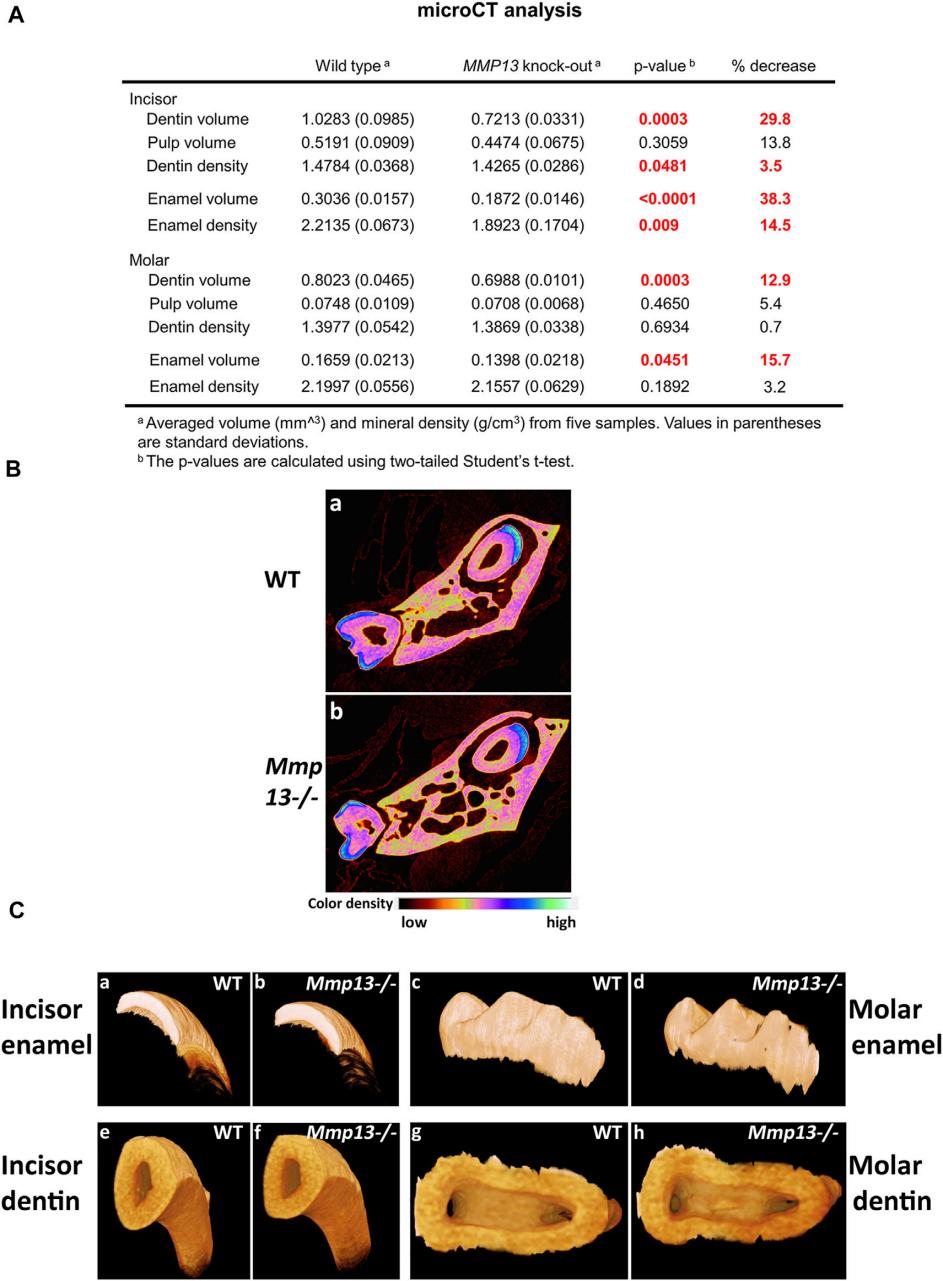
MMP13 deletion reduces dentine enamel volume and density. **(A)** MicroCT analysis of the mandibular first molar and incisor of 3-month-old WT compared with *Mmp13* ^*−/−*^ mice shows a significant decrease in dentin, enamel volume, and mineral density in incisors as well as dentin and enamel volume in molars. **(B)** Densitometry for mandibular incisors and molars in cross section **(a, b)** shows smaller teeth with reduced enamel volume (highlighted blue). **(C)** µCT photographs confirm smaller teeth **(e, f)** with phenotypically altered enamel but no evidence of wear at 3-month **(c, d)**. *n* = 5 for both groups and genotypes.

The maxillary first molar sections from 3-week, 6-week, and 3-month male *Mmp13* ^*−/−*^ and WT mice demonstrated abnormal dentin morphology (Figure 2A). The mineralization front in *Mmp13* ^*−/−*^ was irregular (Figures 2Ab,d,f), the predentin layer was thin and lacked definition, and the odontoblast layer was uneven, less populated, and less well-organized than the regular, organized palisade effect evident in WT samples (Figure 2A). The ordered tubular structure of WT dentin was phenotypically altered and replaced by a disorganized tubular structure at 3-weeks, 6-weeks, and 3-month **(** Figure 2A **)**. The μCT analysis of 3-month-old incisors revealed that the enamel volume was reduced by 38.3% and dentin volume by 29.8% (*p* < 0.0001) in *Mmp13* ^−/−^ samples, with no significant change in body weight and total pulp volume (Figure 3A) or evidence of occlusal wear reported in MMP9-knockout (KO) samples (Figure 3C) (Yuan et al., 2017), indicating the teeth to be smaller. The reduction was also evident in first molars of 3-month-old mice, revealing significant enamel volume reductions of 16% (*p* = 0003) and dentin 13% (*p* = 0.045) **(** Figure 3A **)**. The enamel (14.5% down) and dentin (3.5% down) TMD was significantly reduced (*p* = 0.009; *p* = 0.048 respectively) in mandibular incisors but not molars (enamel *p* = 0.189; dentin *p* = 0.693) (Figures 3A–C).

### MMP13 Promotes Dentinogenesis Repair and Regulates Dystrophic Pulp Mineralization

**FIGURE 4:**
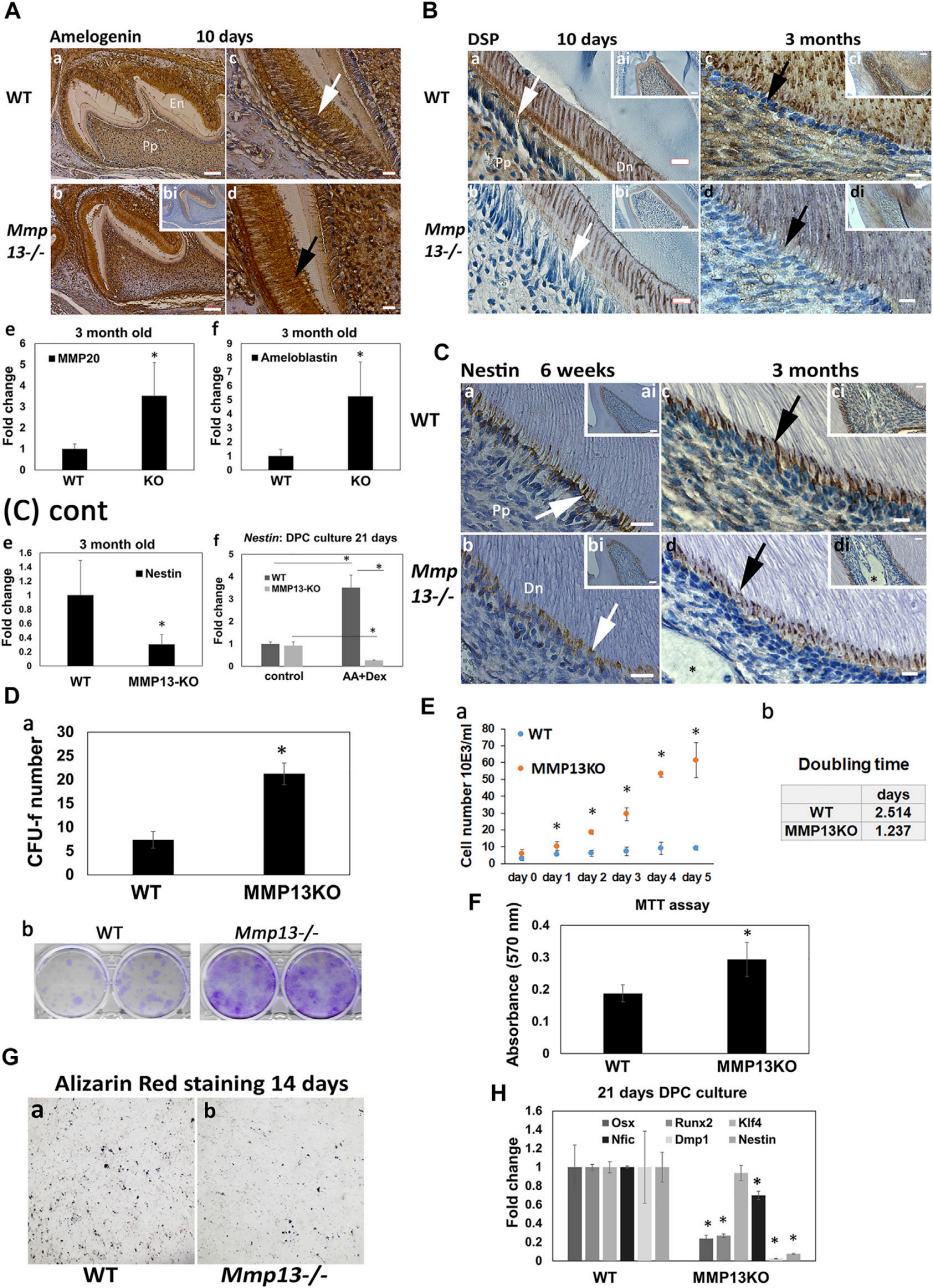
Odontoblast marker expression is reduced after MMP13-loss, as DPC proliferation increases. **(A)** Amelogenin-expression is higher in the ameloblast layer (**c**, white arrow; **d**, black arrow) of *Mmp13* ^*−/−*^ molars thanin WT and unstained control **(bi)** at postnatal 10-day. Enamel volume is also visibly reduced in *Mmp13* ^*−/−*^ compared with WT **(a, b)**. Scale bars = **(c, b)** 100 μm (original mag. ×10); **(c, d)** 10 μm (original mag. ×40). **(e, f)** *Mmp20* and *ameloblastin* gene expression significantly upregulated in tissue from incisors of MMP13KO at 3-month. **(B)** DSP reduced in expression in odontoblast (**a–d**, white and black arrows) and dentin in *Mmp13* ^*−/−*^ sections at 10-day and 3-month **(a–d)**. **(a–d)** represent higher magnifications of the cusp area in inset ai–di. Scale bars = **(a, b)** 7 μm (original mag. ×40); **(ai–di)** 16 μm (original mag. ×20); **(c, d)** 5 μm (original mag. ×63). **(C)** Nestin-expression reduced in *Mmp13* ^*−/−*^ sections **(b, d)** in the odontoblast cell and process (**a–d**, - white, black arrows). Dystrophic calcification not stained by nestin is marked by a black star **(d, di)**. **a–d** represent higher magnifications of the cusp area in inset **ai–di**. Scale bars = **(a–b)** 10 μm (original mag. ×63), **(ai, bi)** 20 μm (original mag. ×20), **(ci, di)** 7 μm (original mag. ×40), and **(c, d)** 5 μm (original mag. ×63). *Nestin* gene expression significantly downregulated in pulp tissue from MMP13KO incisors at 3-months **(e)**, while the DPC culture of tissue from WT and *Mmp13* ^*−/−*^ molars highlights a significant reduction in *nestin* expression in the induced culture at 21-day. **(D) (a)** DPC cultures from WT and *Mmp13* ^*−/−*^ molars were used to determine CFU-F after 14-day, followed by **(D) (b)** Giemsa staining, **(E)** with cell counting performed in regular media with serum up to 5-d **(a)** and cell doubling calculated. **(b) (F)** MTT assay carried out after 48-hr of DPC culture with regular media. **(G)** Five-minute staining time of alizarin red S staining after 14-day in parallel DPC cultures highlights reduced deposits in MMP13KO-cultures. **(H)** qRT-PCR analysis of selected markers cultured with dexamethasone and L-ascorbic acid in DPC from the molars of MMP13KO and WT mice. All *in vitro* experiments based on three independent experiments were carried out in triplicate. Statistical analysis was performed by Student’s t-test for **(Da, F, H)** and one-way analysis of variance (ANOVA) with post hoc Tukey’s for **(Ea)** Abbreviations: Dn, mineralized dentin; Pp, pulp tissue. Sections were examined from six mice at 10-d postnatal and nine mice at 3-month for both genotypes. Data are shown as mean ± s.d., *p < 0.05 versus control.

Pulp tissue responds to injury and irritation by localized inflammation and the production of tertiary dentine, which forms beneath the area of challenge (Lesot et al., 1994; Smith, 2002). There are two types of tertiary dentin formed depending on the severity of the irritating stimulus; mild irritation as a result of cusp damage induces an upregulation of existing odontoblast activity to form reactionary dentine, while stronger stimuli result in odontoblast death and the recruitment of dental pulp progenitor cells, which differentiate into odontoblast-like cells to form reparative dentine (Lesot et al., 1994). In this study, we were interested in the role of MMP13 not only in tooth development, but also in the production of tertiary dentin at the cusp tips of mouse molars. In this study, significant reactionary tertiary dentin was visible at injured and damaged cusp tips in 6-week and 3-month WT mouse molars, but not the *Mmp13* ^*−/−*^ equivalents despite thinner dentin (Figures 3B,C). Dystrophic pulpal-calcification was present more frequently in serial sections of maxillary first molars in *Mmp13* ^*−/−*^ than that in WT (Figure 2C) at 3-week (present 2 out of 5 *Mmp13* ^*−/−*^; 0/5 WT biological samples [*p* = 0.429]), and significantly at 6-week (5/5 *Mmp13* ^*−/−*^; 0/5 WT biological samples [*p* = 0.008]) and 3-month (8/9 *Mmp13* ^*−/−*^; 1/9 WT biological-samples [*p* = 0.003]) (Figure 3C). The dystrophic mineralized-tissue did not exhibit a tubular structure indicative of dentin, and did not stain positively for nestin (Figure 4Cd); however, the marker HDAC5 was expressed in the mineralized tissue (Figure 3Cc), perhaps suggesting a cemental or bony origin (Bradley et al., 2015).

### The Odontoblast Differentiation-Markers, Nestin and DSP, Are Reduced After MMP13-Loss

IHC showed that *in vivo* expression of amelogenin increased in *Mmp13* ^*−/−*^ molar samples at postnatal 10-day compared with WT (Figure 4A), while the gene expression of ameloblastin (>5-fold) and MMP20 (>3-fold) was significantly upregulated at 3-month in DPCs from *Mmp13* ^*−/−*^ incisors (Figures 4Ae,f). DSP expression reduced in *Mmp13* ^*−/−*^ molar dentin and the odontoblast layer at 10-day postnatal, and 3-month samples (Figure 4B), and the preodontoblast-marker nestin was progressively reduced in expression in odontoblasts of *Mmp13* ^*−/−*^ at 6-weeks and 3-month (Figure 4C). qRT-PCR highlighted the significant attenuation of nestin at 3-month in pulp tissue from *Mmp13* ^*−/−*^ mouse incisors (Figure 4Ce), and a (>10-fold) decrease in expression in 21-day *Mmp13* ^*−/−*^ molar-cultures compared with WT (Figures 4Cf,H). The expression of other mineralization markers, dentin matrix acidic phosphoprotein (*Dmp-1*), Runt-related transcription factor 2 (*Runx2*), and osterix (*Osx*) was significantly reduced in DPC cultures from *Mmp13* ^*−/−*^ molars at 21-day (Figure 4H). The Alizarin red staining of DPC cultures from both genotypes demonstrated less calcific deposits at 14-day in knockout-samples (Figure 4G).

DPC proliferation increased after MMP13-loss. The CFU-f assay demonstrated a significant increase in colony formation (>3-fold; *p* < 0.05), which was supported by increased Giemsa staining in MMP13-null cultures (Figure 4D). The cell number was markedly increased at time points up to 5 days (*p* < 0.05) in MMP13KO cultures, with cell doubling time reduced by >50% compared with WT controls (Figure 4E); furthermore, the MTT assay demonstrated a significant increase in cell metabolic activity in MMP13-null cultures (Figure 4F). qRT-PCR showed no difference in the expression of *Klf-4* in DPC *Mmp13* ^*−/−*^ cultures at 21 day (Figure 4H); however, it was significantly upregulated in tissue taken from 3-month old incisor teeth (Figure 7C). *Nifc* was significantly downregulated at 21-day (Figure 4H). Taken together, these results suggest that MMP13 increases dentinogenic differentiation and attenuates DPC proliferation processes.

### MMP13 Ablation Increases Expression of MMPs -8 and 9 *In Vitro* and *In Vivo*

**FIGURE 5:**
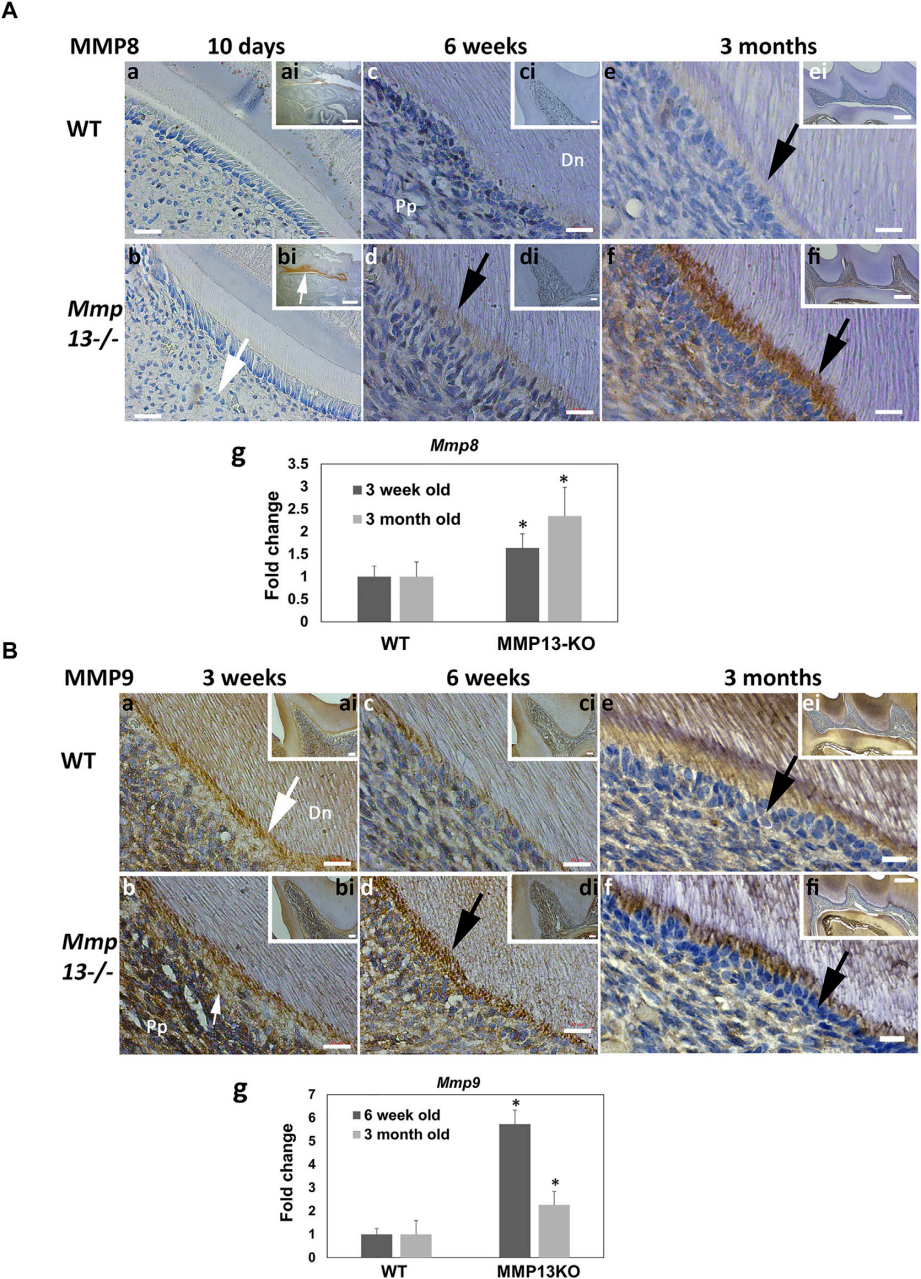
Dentin–pulp expression of other MMPs, MMP-8 and MMP-9, is altered after MMP13-ablation. **(A)** Dental expression of another collagenase, MMP8, was increased in *Mmp13* ^*−/−*^ and mature teeth **(a–f)**. MMP8 expression was low in developing molars (**a, b**—large white arrow) but high in the oral mucosa of MMP13KO (**bi** [inset] white arrow). MMP8 expression progressed in pulp tissue, odontoblast layer, and predentin (**d, f**—black arrows) compared with WT **(c, e)** over time. **c–f** represent higher magnifications of the mesial cusp area of the distal cusp of images inset **ci–fi**. Scale bars = **(a, b)** 5 μm (original mag. ×63); **(ai, bi)** 200 μm (original mag. ×2.5); **(c–f)** 10 μm (original mag. ×63); **(ci, di)** 20 μm (original mag. ×20); and **(ei, fi)** 10 μm (original mag. ×10). qRT-PCR shows increased *Mmp8* expression in pulp tissue from MMP13KO incisors at both 3-week and 3-month **(g)**, while DPC culture of tissue from WT and *Mmp13* ^*−/−*^ mouse molars highlights a significant increase in *Mmp8* expression in mineralization media at 21-day **(h)**. **(B)** Gelatinase MMP9 was evident in odontoblasts, predentin layer, and pulp at 3-week with low expression in mineralized dentin **(a,b)**. Increased MMP9 expression in the odontoblast predentin layer of *Mmp13* ^*−/−*^ molars is highlighted at 3-week **(b)**, 6-week **(d)**, and 3-month **(f)** compared with WT (black, white arrows). **(a–f)** represent higher magnifications of the mesial area of the cusp in inset of **ai–fi**, respectively. Scale bars = **(a–d)** 10 μm (original mag. ×63); **(ai–di)** 20 μm (original mag. ×20); **(e, f)** 5 μm (original mag. ×63); and **(ei, fi)** 100 μm (original mag. ×10). *Mmp9* expression is increased in pulp tissue from MMP13KO incisors at both 6-week and 3-month **(g)**. Abbreviations: Dn, mineralized dentin; Pp, pulp tissue. Sections were examined from six mice at 10-day, 3-week and 6-week and nine mice at 3 months for both genotypes. All qRT-PCR data shown are based on at least three independent biological samples carried out in triplicate, with data shown as mean ± s.d., *p < 0.05 vs. control.

As individual MMPs can synergize and regulate other MMPs activity (Ortega et al., 2003), the effect of MMP13-loss on two highly-expressed dental-MMPs was investigated (Sulkala et al., 2007; Yuan et al., 2017), with the loss of MMP9 previously shown to affect the tooth structure. IHC revealed a high MMP8-expression in the oral mucosa of 10-day teeth, but low dentin–pulp expression. There was an increased expression in the odontoblast layer, predentin, and pulp tissue of *Mmp13* ^*−/−*^ molars at 6-week and 3-month (Figure 5A), which was supported by a significant increase in *Mmp8* expression at 3-weeks and 3-month in pulp tissue from *Mmp13*-null incisors (Figure 5Ag). MMP9 expression decreased in the odontoblast, predentin layer, and pulp of WT compared with *Mmp13* ^*−/−*^ samples at 3-week, with reduced expression in predentin and odontoblast was progressively more evident in WT at 6-week and 3-month (Figure 5B). The mRNA expression of *Mmp9* was increased at 6-weeks and 3-months in MMP13-ablated samples (Figure 5Bg).

### HDAC4 and HDAC5 Show Increased Expression During Mineralization and After MMP13-Loss

**FIGURE 6:**
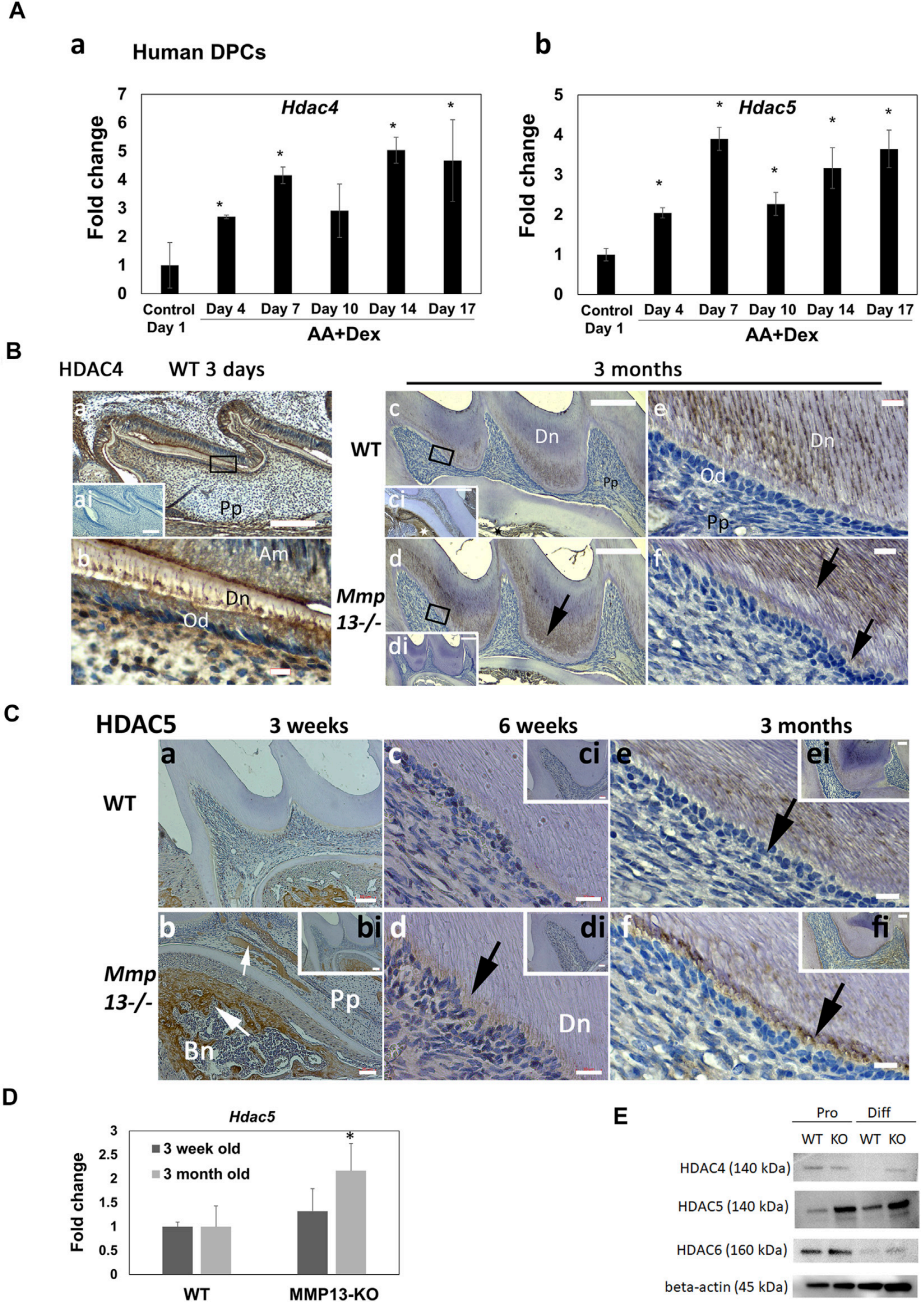
Expression of mineralization-associated HDAC, HDAC4 and HDAC5, in the dentin–pulp complex increased during human-DPC mineralization and after MMP13-loss. **(A)** Time-course human-DPC-culture indicates a progressive significant increase in both *Hdac4* and *5* expressions during mineralization. **(B)** IHC-HDAC4 expression decreases in adults compared with developing teeth **(a–f)**. HDAC4 demonstrated high expression at post-natal 3-day WT samples in the odontoblast layer **(a, b)** compared with unstained control (**ai**—inset). In adult samples, reduced expression of HDAC4 **(c–f)** was demonstrated in pulp, odontoblast **(e, f),** and predentin, with increased expression in mineralized dentin. Higher expression in dentin (**d, f**—black arrows) and odontoblast **(f)** was evident in *Mmp13* ^*−/−*^ samples (black arrows). High HDAC4 expression in adult alveolar bone (**c**- black star; **ci** inset—white star) was evident. **ai** and di represent unstained controls of a and d, while ci is a 10× section of a different area of the same tooth illustrated in C. Scale bars = **(a, ai, c, ci, d, di)** 100 μm (original mag. ×10) and **(b, e, f)** 5 μm (original mag. ×63). **(C)** HDAC5 expression increased in *Mmp13* ^*−/−*^ teeth. HDAC5 expression was low at 3-week in WT and MMP13KO teeth but high in bony and mineralized pulpal deposits evident in *Mmp13* ^*−/−*^ samples [**(b)**, small white arrow] and alveolar bone [**(b)**, large white arrow]. Dentin–pulp HDAC5 expression increased at 6-week **(c, d)** and considerably at 3-month in the pulp, odontoblast, and predentin area of *Mmp13* ^*−/−*^ samples (**d–f**, black arrows). **(b–f)** represent higher magnifications of the indicated area in **(bi–fi)**, respectively. Scale bars = **(a, bi)** 50 μm (original mag. ×10), **(b, ci, di, ei, fi)** 20 µm (original mag. ×20), and **(c, d)** 10 μm **(e, f)** 5 μm (original mag. x 63). Abbreviations: Am, ameloblast; Bn, alveolar bone; Dn, dentin; Pp, pulp. Sections were examined from five mice at 3-day, six mice at 10-day postnatal and nine mice at 3 months for both genotypes. *Hdac5* expression is significantly increased at 3-month in DPC cultures **(D)** from MMP13KO molars by RT-qPCR. **(E)** WB analysis of HDAC4 expression in proliferating mouse DPC cells was similar in MMP13KO and WT but decreased in WT differentiating DPC samples, while HDAC5 increased in differentiating samples and all KO samples supporting data in **(Ca–f)**. HDAC6 expression level was similar in WT and MMP13KO but reduced in differentiating DPCs. All qRT-PCR and WB data shown are based on at least three independent biological samples carried out in triplicate. qRT-PCR data are shown as mean ± s.d., *p < 0.05 vs. control.

MMP13 modulation by the mineralization-associated HDAC-4 (Shimizu et al., 2010) and -5 (Bradley et al., 2015) has been shown in osteoblastic cells. Specifically in DPC cultures, the application of HDACi stimulated pro-mineralization responses by increasing MMP13 expression (Duncan et al., 2016), and broadly the role of acetylation in dentin–pulp hard tissue formation (Yamauchi et al., 2020) has been highlighted. In the current study, the class II HDAC, 4 and 5, expressions significantly increased in mineralizing HDPCs at every time point up to 17-day compared with non-mineralizing control cultures (Figure 6A). Furthermore, IHC-analysis highlighted the high HDAC4-expression in developing odontoblasts, ameloblasts, alveolar bone, dentin, and pulp at post-natal 3-day WT samples compared with unstained control, while in adult teeth, HDAC4-expression was greatly reduced, but was higher in *Mmp13* ^*−/−*^ samples compared with WT (Figure 6B). *In vivo* HDAC5-expression was generally low in WT pulp and the odontoblast layer, but high in alveolar bone (Figure 6Cb), with notable expression in the odontoblast and predentin layer of 3-month *Mmp13* ^*−/−*^ molars compared with controls (Figure 6C). HDAC5’s high expression in the alveolar bone agreed with a previously reported positive control (Figure 6Cb) (Bradley et al., 2015). An Increased *Hdac5* mRNA expression was evident at 3-month in pulp tissue from *Mmp13* ^*−/−*^ incisors compared with WT (Figure 6D). The Western blot (WB) analysis of HDAC4 expression in cultured pulp tissue revealed that expression was stable under proliferating (5-day culture) and differentiating (14-day culture) conditions in *Mmp13* ^*−/−*^ samples; however, HDAC4 expression decreased in the differentiating cells of WT pulp tissue under mineralizing compared to proliferating conditions (Figure 6E). HDAC5 increased in differentiating DPC samples and in MMP13KO samples in both proliferating and differentiating conditions compared to WT, supporting data in (C). The Class IIb HDAC6, showed a significant reduction in expression under mineralizing conditions with a slight increase in MMP13KO samples compared with WT (Figure 6E). It is clear that selected mineralization-associated class II HDACs are altered in expression in mineralizing conditions (Huynh et al., 2016), and also the novel finding suggests that MMP13 influences their expression in the dentin–pulp complex.

### MMP13-Loss Significantly Altered DPC Gene Expression at 3 Months Reducing Mineralization-Associated Differentiation Genes But Increasing the Expression of Proliferation-Related Genes

To determine the molecular mechanisms whereby MMP13 alters mineralization effects *in vitro* and *in vivo*, we performed RNAseq analysis. Based on initial qRT-PCR experimentation on pulp tissue isolated from WT and *Mmp13* ^*−/−*^ (Figure 4H), we hypothesized that MMP13-loss would reprogram gene expression during mineralization, altering the expression of a range of genes, while decreasing the expression of specific mineralization-associated markers. Subsequently, we isolated mRNA from the incisor teeth of WT and *Mmp13* ^*−/−*^ mice at 3-months and showed that of the 45,796 genes analyzed, MMP13-loss increased the expression > two-fold of 3,352 transcripts and suppressed the expression of 1,668 genes, representing alteration of 10.96% of the oligonucleotides (Table 1). The top 20 most up- and downregulated genes at 3-month are listed in (Supplementary Tables S2, S3). The observed gene expression patterns demonstrate that although MMP13-loss induced transcriptional change, more genes were induced than suppressed and the expression of selected genes associated with energy and metabolism (Cartpt 365.4-fold up (Takatani et al., 2021)) and development (Duxf3, 136.6-fold up (Chen and Zhang, 2019)) was dramatically upregulated (Supplementary Table S2), while downregulation was at a more modest level (Supplementary Table S3). Notably, similar patterns of differential gene expression have been reported in other MMP13KO high-throughput transcriptomic studies albeit using microarray technology in different tissues (Toriseva et al., 2012). Interestingly, in support of the hypothesis that showed the absence of MMP13 reduced the differentiation of odontoblasts and increased cell turnover, selected mineralization-associated transcripts were seen to be significantly downregulated (*Coll-1*, 4.2-fold down; *TGFβ1*, 3.45-fold down), while other markers of proliferation were upregulated (*Pcna*, 2.7-fold up; *Ki67* 4.95-fold up). The downregulation of the odontoblast-marker *nestin*, and the upregulation of several *Mmps* (*-8*, *-9* and *-20*), identified in the RNAseq was validated to be of similar magnitude in subsequent qRT-PR experimentation (Table 2). In order to further interrogate mechanistic interactions, pathway analysis was investigated.

**TABLE 1:** Number of genes demonstrating >1 and >2 absolute fold change in *Mmp13* ^*−/−*^ compared with WT murine dental pulp cell cultures. Genes that were differentially expressed (>1.0 and >2.0 absolute fold change) in *Mmp13* ^*−/−*^ relative to WT control were identified after passing the t-test (*p* < 0.05) and post hoc multiple test (Storey with bootstrapping) with a corrected *q* value of 0.05.

De-regulated gene *q* < 0.05	Total number of genes	Upregulated	Downregulated	Genes altered as (%)of total genes present (45,796)
Absolute FC > 1.0	8,228	4,314	3,914	17.96
Absolute FC > 2.0	5,020	3,352	1,668	10.96

**TABLE 2:** Comparison of RNA-sequencing and qRT-PCR from dental pulp cells of *Mmp13* ^*−/−*^ compared with WT at 3 months. qRT-PCR was averaged from three independent biological experiments carried out in triplicate. Fold change represents an average value for qRT-PCR.

Gene name	Absolute fold change RNA-seq in *Mmp13* ^*−/−*^	Absolute fold change qRT-PCR in *Mmp13* ^*−/−*^	HGNC gene ID
*Nestin*	2.18 down	3.5 down	18008
*Mmp8*	7.84 up	2.25 up	17394
*Mmp9*	1.52 up	2.1 up	17395
*Mmp20*	2.89 up	3.3 up	30800
*Ameloblastin*	2.26 up	5.2 up	11698

### MMP13-Ablation Alters Multiple Pathways, Downregulating the Wnt Signaling and Pluripotency Pathway and Decreasing in Axin2 *In Vivo*

**FIGURE 7:**
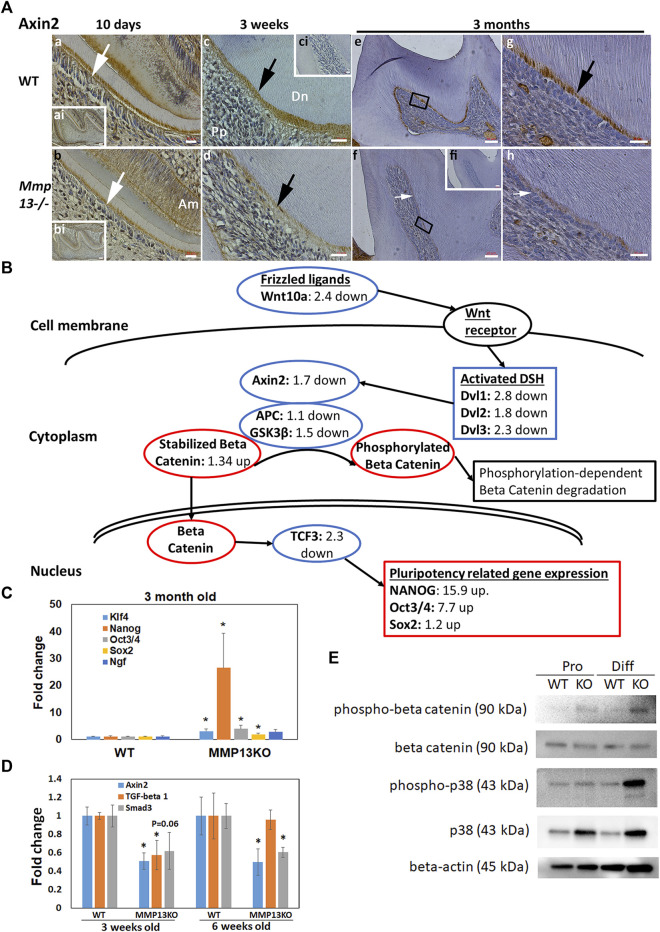
Wnt responsive gene *Axin2* expression is reduced in *Mmp13* ^*−/−*^ odontoblasts, while Nanog is increased and the Wnt signaling pathway suppressed after MMP13-loss. **(A)** IHC-Axin2 expression is decreased in the odontoblast and predentin layer of developing (10-day [**(a, b)**—white arrow], 3-week [**(c, d)**—black arrow]), and mature first molars (3-month [**(e–h)** white, black arrow]) of *Mmp13* ^*−/−*^ samples compared with WT samples. **(ci)** represents an unstained control of the same cusp as **(c)**, while **(g, h)** are higher magnifications of the indicated area identified in **(e, f)**, respectively. **(a, b)** represent higher magnifications of the indicated area in the **(ai, bi)**, respectively. Scale bars = **(ai, bi, ci, e, f)** 20 μm (original mag. ×20), **(a–d)** 10 μm (original mag. ×40), and **(g, h)** 5 μm (original mag. ×63). Abbreviations: Am, ameloblast; Dn, dentin; Pp, pulp. Sections were examined from five mice at 3-day, six mice at 10-day postnatal, and nine mice at 3 months for both genotypes. **(B)** Selected markers in the Wnt signaling and pluripotency pathway colored and labeled to reflect RNA-sequencing gene expression changes in tissue taken from *Mmp13* ^*−/−*^ teeth compared with WT controls at 3 months. Pathway analysis (Pathvisio version 3.3.0+), after accounting for redundancy, highlighted a significant downregulation in the Wnt signaling and pluripotency pathway after MMP13-loss with 97 (88%) genes altered, a z-score of 3.33, and a *p* value of <0.0001 with a permutation test. Marks of interest that have been previously related to Wnt signaling in teeth (*Wnt10a*, *GSK3β*, and *activated DSH*) and odontoblasts (*Axin2*) and reduced in expression are highlighted. **(C)** Expression of *Nanog* (>25 fold), *Klf4*, *Pou5f1 (Oct3/4)*, *and Sox2* is significantly increased in pulp tissue taken from incisor teeth of MMP13KO mice at 3 months compared with WT, which validates the results of the RNA-sequencing in **(B)**. **(D)** *Axin2* significantly reduced in expression at 3 and 6 days, *Tgfβ1* at 3 days, and *Smad3* at 6 days in pulp tissue from the incisors of MMP13KO compared with WT, corroborating pathway analysis in **(B)** and Supplementary Table S5. **(E)** WB analysis of phospho-β-catenin expression highlights increases in MMP13KO in both proliferating and differentiating DPCs, while β-catenin appears similarly expressed in all groups. Phospho-p38 increased in MMP13KO in differentiating DPCs, not proliferating, while p38 increased in the KO sample under both conditions. RNA-sequencing experiment is based on three independent biological replicates for both genotypes, while qRT-PCR experimentation carried out in four independent replicates for both genotypes in triplicate, and WB data shown are based on at least three independent biological samples carried out in triplicate. Statistical analysis was carried out by the student t-test with data shown as mean ± s.d., *p < 0.05 versus control.

To elucidate the biological processes and identify potential pathways that are affected by MMP13 in primary murine dental pulp tissue at 3-month, the RNAseq gene expression dataset was subjected to pathway analysis using Go-Elite and Pathvisio analysis software (Duncan et al., 2016). Bioinformatics analysis using strict redundancy controls revealed the significant upregulation (Supplementary Table S4) and downregulation (Supplementary Table S5) of multiple pathways related to the host of metabolic and immunological pathways. The subsequent analysis focused on pathways that have previously been identified as being central to developmental and repair processes in the odontoblast cell and dentin–pulp complex in general. A significant downregulation of the Wnt-signaling and pluripotency pathway (<0.001) and the MAPK pathway (<0.003) was specifically noted as Wnt has previously been strongly linked to the odontoblast differentiation (Kawata et al., 2021) and tooth development (Kim et al., 2021), while upregulating of the MAPK pathway has been linked to DPSC differentiation (Lv et al., 2016) and tooth development (Chen et al., 2018). The pathway illustration (Figure 7B) highlighted the dysregulation of several key markers in the Wnt signaling and pluripotency pathway with significant increases in the pluripotency markers *Nanog* (15.9-fold up) and *Oct3/4* (7.7-fold up), findings that were validated in subsequent qRT-PCR (Figures 7B,C). Furthermore, RNAseq at 3-month revealed that mineralization markers associated with DPC differentiation were decreased in expression in *Mmp13* ^*−/−*^ including the odontoblast-responsive Wnt gene *Axin2* (2.5-fold down), *Smad3* (2.15-fold down), and *TGFβ1* (3.45-fold down) (Figure 7B); findings that were significantly verified by qRT-PCR at 3 and 6 weeks (Figure 7D). From this group of genes, *Axin2* was further evaluated by IHC as it is expressed in differentiating odontoblasts (Bae et al., 2015), and has previously been implicated with Wnt/β-catenin signaling in odontoblast development and maturation (Lohi et al., 2010; Babb et al., 2017). Furthermore, Axin2 is considered a direct target of canonical Wnt signaling (Kim and Simmer 2007). In this study, Axin was reduced in expression in *Mmp13* ^*−/−*^ teeth *in vivo* at 10-day, 3-week and 3-month in the odontoblast and predentin central pulp tissue compared with WT and negative controls (Figure 7A), confirming the interaction between MMP13 and Wnt signaling in the control of DPC differentiation evidenced in the RNAseq pathway analysis (Figure 7B). The links between MMP13 expression and Wnt/β-catenin signaling have been reported elsewhere in mechanically stretched chondrocytes (Song et al., 2021), and between MMP9, 13, and Wnt in maxillary-expansion techniques (Guerrero et al., 2020). Further analysis revealed that although β-catenin expression was similar between WT and MMP13 samples, phospho-β-catenin protein expression was increased in DPCs from MMP13KO cultured in proliferating and markedly in differentiating conditions, which fits with phosphorylation-dependent degradation of β-catenin is a key step in turning off Wnt signals (Figure 7E). Although the MAPK pathway was significantly downregulated (Supplementary Table S5), the protein expression of p38 and phosphorylated-p38 was increased in MMP13KO under mineralizing conditions, a finding linked to enhanced odontogenic differentiation *in vitro* (Du et al., 2020) with phosphorylation linked to odontoblast-stimulation *in vivo* (Simon et al., 2010) (Figure 7E). Despite increases in phosphorylated p38, there was reduced dentine and altered dentin structure in this study, which suggests the absence of MMP13 in tooth development and repair may dysregulate the normal odontoblast activity, which has been demonstrated with other MMPs in tooth development (Yuan et al., 2017).

## Discussion

Our initial aim was to determine whether MMP13 contributed to tooth development and secondary and reactionary tertiary dentinogenesis. To investigate this, we used an *Mmp13^−/−^* mouse model, which exhibits a normal lifespan, and has no major phenotypic abnormalities (Takaishi et al., 2008). We demonstrated that *Mmp13* ^*−/−*^ mice with no change in body-weight or pulpal volume, exhibit an abnormal dental-phenotype, with distorted dentin structure and reduced dentin volume and density but without occlusal wear reported in MMP9-KO samples (Yuan et al., 2017). These novel results support experimentations using Mmp13^−/−^ mice in other mineralizing-tissues, in which ossification was delayed, collagen accumulated in the growth plate region of long bones (Yamagiwa et al., 1999; Inada et al., 2004), and bone healing was impaired after fracture (Behonick et al., 2007). MMP13 is likely to have additional reparative roles in the dentin–pulp complex, beyond regulating tooth developmental and dentinogenesis, by cleaving bioactive growth factors (GFs) or assisting in the dentin matrix component release to stimulate cell migration and differentiation (Ortega et al., 2003; Okamato et al., 2018). The fact that MMP13-polymorphisms are linked epidemiologically to decreased caries-resistance (Tannure et al., 2012; Vasconcelos et al., 2019), hints at a range of potential mechanisms including defective dentin development (Vasconcelos et al., 2019). MMP-13 expression has previously been linked to the promotion of regenerative responses (Toriseva et al., 2007) and differentiation (Lei et al., 2013) in certain cell types, with MMP-13 protein expression increased in mineralizing cultures, as described in other studies (Winchester et al., 1999; Suri et al., 2008; Duncan et al., 2016) as well as in this study *in vivo* (Figure 1).

Within this study, the observed dental phenotype in *Mmp13* ^*−/−*^ mice had similarities to certain hereditary dentin-defects, traditionally subdivided into three types of dentinogenesis imperfecta (DI) and two of dentin dysplasia (Kim and Simmer 2007). DI type-III is well characterized phenotypically and linked to the mutation in the *Dspp* gene (von Marschall et al., 2012), demonstrating a widened predentin layer, reduced dentin thickness and enlarged pulp chamber (Kim and Simmer 2007). Notably, studies investigating tooth development in *Dspp* ^*−/−*^ (Sreenath et al., 2003), *Dmp-1* ^*−/−*^ (Ye et al., 2004), and *Mmp9* ^*−/−*^ (Yuan et al., 2017) mice, reported developmental effects similar to DI type-III. It has been suggested that this altered phenotype may be linked to a disturbance in the ordered secretion of extracellular matrix (ECM) by odontoblasts and its subsequent maturation and degradation (Ni and Chen 2014; Yuan et al., 2017) with a critical role for collagenases like MMP13, in organizing ECM deposition, facilitating differentiation, and GF-cleavage from ECM stores suggested (Ortega et al., 2003; Nkyimbeng et al., 2013). To offer insight into MMP13’s role in tooth mineralization processes *in vivo*, a time-course was carried out revealing high-expression of MMP13 in the odontoblast layer and predentin layer in teeth particularly during primary dentinogenesis, which reduced in secondary dentinogenesis (Figure 1). Although our results also exhibit the irregular mineralization front and altered tubule regularity described in DI type-III (Figure 2A), the appearance resembles other dentin-developmental defects and *Tgfbr2*-deletion (Ahn et al*.*, 2015), rather than DI type-III with a normal pulp chamber size and increase in pulpal-calcification. The observation of frequent dystrophic pulp-mineralization in *Mmp13* ^*−/−*^ teeth, suggests that MMP13 may have a role in the controlled regulation of ECM turnover and organization of mineralization in the dentin–pulp complex. Indeed, the mineralization morphologically resembled multiple discrete calcifications (Figure 2C) rather than a widespread dystrophic calcification previously reported (Kim and Simmer 2007; Ahn et al., 2015). Tertiary reactionary dentin at the cusp-tips was notably reduced in *Mmp13* ^*−/−*^ compared with WT, supporting a potential role for MMP13 in the ordered reparative mineralization processes in response to occlusal irritation (Figure 2B). Previously, MMP13 was increased in dentin in response to caries lesions, highlighting a potential defensive role in regulating tertiary dentinogenesis (Loreto et al., 2014). The formation of reparative dentine is regulated by bioactive molecules, including bone morphogenic proteins, growth factors, and MMPs which are “fossilized” in the dentine matrix (Cassidy et al., 1997; Grando Mattuella et al., 2007) prior to being released by caries, trauma, or by dental materials (Graham et al., 2006; Tomson et al., 2007). Identifying specific key mediators, like MMP13, that promote the production of tertiary defensive dentin, not only has scientific but clinical relevance as the deposition and quality of the tertiary dentin response in response to injury increase the thickness of dentin between the injury and the pulp, helping to preserve pulp vitality (Smith, 2002).

Although enamel-formation was not the primary focus of this work, MMP13 was strongly expressed in the ameloblast layer of developing teeth (Figure 1 **)** with histology and µCT analysis highlighting significant alterations in the enamel volume and density in *Mmp13* ^*−/−*^ teeth and an increase in amelogenin expression, but no increase in occlusal wear. MMP20 has previously been shown to regulate mineralization by cleaving amelogenin during the growth of enamel-like crystals (Kwak et al., 2016; Prajapati et al., 2018). MMPs also facilitate cell movement during development and considerable attention has been directed at the role of MMP20 in facilitating ameloblast movement (Bartlett and Smith, 2013); however, for the first time *in vivo* MMP13-expression is identified in early dentin mineralization and throughout the secretory stages of amelogenesis (Figure 1A). These results are suggestive of a potentially novel role for MMP13 in processing and degrading enamel proteins during development (Simmer and Hu 2002).

Micro-CT analyses demonstrated that teeth were significantly smaller in *Mmp13* ^*−/−*^ mouse, which could have been attributed to a general developmental problem in the *Mmp13* global KO phenotype; however, animal body-weight was unaltered between control and WT. Greater volumetric changes evident in incisors compared with molars may reflect the continual growth pattern evident in rodent incisors, which although not reflective of human teeth, may highlight an accumulative deficiency in dentinogenesis and amelogenesis in *Mmp13*-null mice. Odontoblast-activity was assessed in *Mmp13* ^*−/−*^ teeth with *nestin* levels significantly downregulated both *in vivo* and *in vitro* and DSP reduced at both developmental and adult time-points (Figure 4B). DSP and dentin phosphoprotein (DPP) are two of the principal components of non-collagenous dentin matrix (Butler et al., 2002), with research highlighting that DSP is processed by MMP9 both *in vitro* and *in vivo* (Yuan et al., 2017); this suggests a possible role for others MMPs, such as MMP13 in DSP processing and mineralization processes in dentin (Okamoto et al., 2018). Our group has already identified that *Mmp9* mRNA expression increased in the bone after MMP13-deletion and in DPC-cultures after pharmacological MMP13-inhibition (Duncan et al., 2016; Nakatani et al., 2016). MMPs form an intricate support network, with feedback and synergy regulating other MMPs activity critical for maintaining tissue homeostasis (Ortega et al., 2003). Notably, MMP9 and MMP13 have been highlighted in the bone to work synergistically in remodeling and development (Engsig et al., 2000). In order to investigate this, the expression of the most common dentin collagenase, MMP8 (Sulkala et al., 2007) and the gelatinase MMP (-9), whose loss is known to affect the dental structure (Yuan et al., 2017), these markers were investigated to confirm that the observed effects of MMP13 were not partly attributable to MMP8 or MMP9-knockdown or rescue. Both MMP8 and MMP9 demonstrated an increased gene and protein expression in the predentin layer of knockout mice molars, corroborating a role for feedback and compensation within the collagenases and wider MMP-family (Figure 5).

Research from our group and others linking MMP13 with class IIa mineralization-associated HDACs in *in vitro* and in the bone (Shimizu et al., 2010; Wein et al., 2015; Duncan et al., 2016; Nakatani et al., 2016), prompted an investigation into the dental HDAC4/5 expression *in vivo*. Previous IHC-analyses in third molars highlighted odontoblastic HDAC-expression and that class-II HDAC partly regulates the odontogenic-related gene expression (Klinz et al., 2012). In this study, IHC-analysis confirmed a likely role for HDAC4 in primary dentinogenesis with high-expression evident in active young odontoblasts and pulp cells, but not in mature odontoblasts. From a translational perspective, HDAC4 had a high gene expression in mineralizing human-DPCs, while protein expression in differentiating mouse DPCs reduced, a finding reversed after MMP13-ablation (Figure 6), with links with previous work using osteoblastic cells highlighting that HDAC4 binds to the MMP13 promoter to alter transcription (Shimizu et al., 2010). Previously, *Hdac4* ^*−/−*^ mice exhibited normal dentin morphology, but shorter root length (Ono et al., 2016). In this study, HDAC5-expression was low in developing teeth; however, expression notably increased in the odontoblast layer of MMP13-deleted samples and in differentiating MMP13KO DPCs cultures, suggesting potential mechanistic roles for HDAC5 in regulating odontoblast differentiation as demonstrated elsewhere in osteoblasts (Kang et al., 2005) (Figure 6). Unlike class IIa HDAC, the class IIb, HDAC6 did not show altered expression after MMP13-deletion, but was significantly reduced in expression in differentiating compared with proliferating DPC cultures (Figure 6).

An increase in DPC-proliferation in *Mmp13* ^*−/−*^ cultures was notably accompanied by downregulation of a range of dentinogenic differentiation-markers, *in vivo* (Nestin and DSP) and *in vitro* including *Osx*, *nestin*, and *Runx2* in mineralizing DPCs. Runx2 is a key transcription factor that regulates osteoblast cell differentiation and also binds to the promoters of several genes that regulate differentiation during mineralization, including *Dmp-1* which was also downregulated in *Mmp13*-null cultures. Previous *in vitro* cancer research has highlighted varied effects of MMPs on DPC-proliferation with MMP7 inhibiting cell proliferation (Zhang et al., 2014) and MMP9-inhibition increasing proliferation of satellite cells in dystrophic-muscle (Hindi et al., 2013). Mechanistically, MMP-control of cell division and proliferation has been based on the regulation of GF-availability and the activation/inactivation of GF-receptors (Rodríguez et al., 2010). Research from our group has highlighted that pharmacological MMP13-inhibition reduced cell migration; however, the effects on DPC proliferation were not previously investigated (Duncan et al., 2016). RNAseq analysis revealed a significant upregulation in *Mmp13* ^*−/−*^ DPC cultures of the liked self-renewal and pluripotency factors (Fong et al., 2008), *Nanog* (15.9 fold up), *Oct3/4* (7.7 fold up), and *Sox2* (1.2 up), a finding that was validated with the addition of *Klf4* in tissue from 3-month old mouse pulp. *Klf4* is involved in a range of cellular processes including cell growth, proliferation, and differentiation and has previously been shown to be involved in dentinogenesis and odontoblastic differentiation via histone acetylation (Tao et al., 2019). Within this study, *Klf4* expression was not significantly changed in the comparative DPC culture, but was increased in tissue harvested from incisor teeth at 3-month, a finding which can likely be attributed to the unique characteristics of mouse incisor teeth in which the DPCs continue to proliferate as the tooth erupts throughout life. The expression of *Nanog* (Pitrone et al., 2019) and *Sox2* (Liu et al., 2015) has been linked to increased proliferation is dental pulp and other tissues, while *Oct3/4* expression has been shown to alter cell proliferation during tooth morphogenesis (Nakagawa et al., 2012), as well as controlling differentiation processes.

The mechanisms driving dentine matrix mineralization and MMP13’s role in promoting mineralization, tooth development, and dentinogenesis have not been previously elucidated. Using high-throughput RNAseq analysis, this study clearly demonstrates the MMP13-ablation induced the differential expression of several novel genes in pulp tissues from MMP13KO and WT incisor teeth. The involvement of many unidentified transcripts has not previously been demonstrated in other MMP13 studies (Toriseva et al., 2012; Zhang et al., 2012; Supplementary Tables S2, S3); however, similarly, to previous *Mmp13* ^*−/−*^ high-throughput array studies, genes were both induced and suppressed by after ablation (Toriseva et al., 2012). Notably, in this study more genes were significantly dysregulated (5,000+ >2.0 fold) even with strict statistical processing perhaps highlighting tissue-dependent differences in MMP13 effects (Table 1). Notably, high-throughput analysis demonstrated the alteration of several gene pathways (Supplementary Tables S4, S5), with upregulation associated with alterations in biological areas such as metabolism and immunological processes, including “Glucocorticoid and Mineralocorticoid Metabolism,” “Macrophage markers,” “Cytokines and Mineralization response” and the “Osteoclast” pathway; this supports previous research on MMP13-deficient mice which has highlighted increases in inflammation in the lung (Sen et al., 2010) and altered the expression of several inflammatory markers in granulation tissue (Toriseva et al., 2012). In relation specifically to downregulated pathways, immunological pathways were also significantly altered (TNF-alpha NF-kB signaling pathway, IL-6 signaling pathway, and IL-3 signaling pathway)**,** while other pathways previously related to odontoblast function and development were also altered including the Wnt-signaling pathway (Lohi et al., 2010; Babb et al., 2017) and the MAPK signaling pathway (Simon et al., 2010; Du et al., 2020).

Of these, the Wnt signaling pathway (Figure 7) was of particular interest due to its critical role in tooth development and odontoblast function (Lohi et al., 2010), with *Axin2* and *Wnt10a* significantly downregulated in MMP13KO samples, both of which have previously been highlighted to be reduced in the odontoblasts of malformed crowns in Wnt-less mice (Bae et al., 2015). Wnt10a expression has been shown to induce DSP expression and odontoblast differentiation (Yamashiro et al., 2007), while Axin2 expression in odontoblasts has been shown to enhance reparative dentinogenesis (Babb et al., 2017) and root thickness (Zhang et al., 2021) and has implicated Wnt/β-catenin signaling in odontoblast development and maturation (Lohi et al., 2010; Babb et al., 2017). Axin2 is a direct target of canonical Wnt signaling (Kim and Simmer 2007) and is seen in a restricted pattern during mouse embryogenesis and organogenesis with reports suggesting that Axin2 participates in a negative feedback loop that limits the duration of a Wnt-initiated signal (Jho et al., 2002). *Axin2* alteration by the RNAseq and Axin2 increase in WT by IHC clearly demonstrates the link between Wnt/β-catenin signaling and MMP13 expression in the dentin–pulp complex and highlights a mechanism by which *Mmp13* can modify the odontoblast activity and subsequent dentin formation.

In conclusion, the *in vitro* and *in vivo* evidence presented here demonstrates that MMP13 plays an important role in multiple functions critical to the regulation of tooth development, odontogenic differentiation, and dentin–pulp reparative mechanisms. MMP13 absence altered dentine quality and volume and the deposition of defensive tertiary reactionary dentine while increased dystrophic mineralization highlighted a key role for MMP13 in the organizing and regulation of tooth development and dentin–pulp regeneration. In addition, the results of the present study show mechanistic links to class IIa HDAC expression and the β-catenin-dependent Wnt-signaling pathway for the physiological function of MMP-13 in developing teeth and identified potential novel targets for the development of next-generation therapies for the promotion of improved reparative responses in the damaged dental pulp.

## Data Availability

The original contributions presented in the study are publicly available. This data can be found here: https://www.ncbi.nlm.nih.gov/geo/query/acc.cgi?acc=GSE178898.
