# Supplementary material for: The Critical Role of MMP13 in Regulating Tooth Development and Reactionary Dentinogenesis Repair Through the Wnt Signaling Pathway
Source: Front Cell Dev Biol. 2022 Apr 21;10:883266. doi: 10.3389/fcell.2022.883266 (PMC9068941; doi:10.3389/fcell.2022.883266)
Supplement: Supplementary file 1 [file Table1.DOCX]

**Supplementary Table 1** Primer sequences for qRT-PCR

| **Gene** | **Primer sequence (5’ to 3’)** |
| --- | --- |
| **Mouse Sp7 transcription factor 7 (Osterix)** | (F)- AGAGGTTCACTCGCTCTGACGA  (R)- TTGCTCAAGTGGTCGCTTCTG |
| **Mouse runt related transcription factor 2 (Runx2)** | (F)- CCTGAACTCTGCACCAAGTCCT  (R)- TCATCTGGCTCAGATAGGAGGG |
| **Mouse Kruppel-like factor 4 (Klf4)** | (F)- CTATGCAGGCTGTGGCAAAACC  (R)- TTGCGGTAGTGCCTGGTCAGTT |
| **Mouse nuclear factor I/C (Nfic)** | (F)- AGTGTCACGGAGCTGATCCAAG  (R)- CTGGGTAGAGTCCTTCTCATGC |
| **Mouse Nestin** | (F)- CCCTGAAGTCGAGGAGCTG  (R)- CTGCTGCACCTCTAAGCGA |
| **Mouse Dmp1** | (F)- GAAAGCTCTGAAGAGAGGACGG  (R)- CCTCTCCAGATTCACTGCTGTC |
| **Mouse Hdac5** | (F)- ACCAGCAGTTCCTGGAGAAGCA  (R)- TCCGTCAGCTCCTCTTCTGTCT |
| **Mouse MMP8** | (F)- GATGCTACTACCACACTCCGTG  (R)- TAAGCAGCCTGAAGACCGTTGG |
| **Mouse MMP9** | (F)- GCTGACTACGATAAGGACGGCA  (R)- TAGTGGTGCAGGCAGAGTAGGA |
| **Mouse MMP13** | (F)- GATGACCTGTCTGAGGAAGACC  (R)- GCATTTCTCGGAGCCTGTCAAC |
| **Mouse MMP20** | (F)- GAAGTGGCTGAACGAGGCATTG  (R)- TTGTCCGTGGAGGACCTTGCAT |
| **Mouse Ameloblastin** | (F)- TCCGAAAACCCACCAACACCTG  (R)- AGCGGATGCTTTGTTGTGTGCC |
| **Mouse Oct3/4 (Pou5f1)** | (F)- GTTGGAGAAGGTGGAACCAA  (R)- CTCCTTCTGCAGGGCTTTC |
| **Mouse Sox2** | (F)- TCCAAAAACTAATCACAACAATCG  (R)- GAAGTGCAATTGGGATGAAAA |
| **Mouse Nanog** | (F)- GGTTGAAGACTAGCAATGGTCTGA  (R)- TGCAATGGATGCTGGGATACTC |
| **Mouse NGF** | (F)- GTTTTGCCAAGGACGCAGCTTTC  (R)- GTTCTGCCTGTACGCCGATCAA |
| **Mouse Axin2** | (probe) 56-FAM/TTCCACAGG/ZEN/CGTCATCTCCTTGG/3IABkFQ/  (primer 1)- CTTTCCAGCTCCAGTTTCAGT  (primer 2)- AGTGTCTCTACCTCATTTTCCG |
| **Mouse TGFβ1** | (probe) 56FAM/ATAGATGGC/ZEN/GTTGTTGCGGTCCA/3IABkFQ/  (primer 1)- CCGAATGTCTGACGTATTGAAGA  (primer 2)- GCGGACTACTATGCTAAAGAGG |
| **Mouse SMAD3** | (probe) 56FAM/CAGCCGACC/ZEN/ATCCAGTGACCT/3IABkFQ/  (primer 1)- GCGGCAGTAGATAACGTGAG  (primer 2)- GAACACCAAGTGCATTACCATC |
| **Mouse beta-actin** | (F)- TCCTCCTGAGCGCAAGTACTC  (R)- CGGACTCATCGTACTCCTGCTT |
| **Human MMP13** | (F)- CCTTGATGCCATTACCAGTCTCC  (R)- AAACAGCTCCGCATCAACCTGC |
| **Human MMP8** | (F)- CAACCTACTGGACCAAGCACAC  (R)- TGTAGCTGAGGATGCCTTCTCC |
| **Human Hdac4** | (F)- AGGTGAAGCAGGAGCCCATTGA  (R)- GGTAGTTCCTCAGCTGGTGGAT |
| **Human Hdac5** | (F)- CGCTGA GAATGGCTTTACTGGC  (R)- GTGTAGAGGCTGAACTGGTTGG |
| **Human beta-actin** | (F)- CACCATTGGCAATGAGCGGTT  (R)- AGGTCTTTGCGGATGTCCACGT |

**Supplementary Table 2** Top 20 known genes upregulated in dental pulp cell cultures of *Mmp13*^-/-^ mice. Normalization of three independent biological replicates in each group was carried out using the DESEQ method with a threshold of one. Genes that were differentially expressed (>2.0 absolute fold change, >1 log_2_) in the *Mmp13^-/-^* relative to WT control were identified after passing a t-test (p<0.05), post-hoc multiple test (Storey with Bootstrapping) with a corrected q value of 0.05.

| **Gene** | **Gene name** | **Fold change** | **q value** |
| --- | --- | --- | --- |
| CART prepropeptide | Cartpt | 365.4 | 0.0027 |
| Double homeobox family member 3 | Duxf3 | 136.6 | 0.0135 |
| Eosinophil-associated, ribonuclease A family, member 2 | Ear2 | 101.6 | 0.0187 |
| Eosinophil-associated, ribonuclease A family, member 1 | Ear1 | 75.5 | 0.0264 |
| WAP four-disulfide core domain 21 | Wfdc21 | 67.6 | 0.0121 |
| Ribonuclease P RNA-like 2 | Rprl2 | 62.3 | 0.0027 |
| Eosinophil-associated, ribonuclease A family, member 6 | Ear6 | 53.1 | 0.0247 |
| Mucin 5, subtypes A and C, tracheobronchial/gastric | Muc5ac | 51.0 | 0.0176 |
| One cut domain, family member 3 | Onecut3 | 44.5 | 0.0103 |
| CD69 antigen | Cd69 | 44.5 | 0.0094 |
| Proteoglycan 3 | Prg3 | 44.4 | 0.0254 |
| Sodium channel, voltage-gated, type X, alpha | Scn10a | 40.7 | 0.0192 |
| cDNA sequence AY702103 | AY702103 | 40.1 | 0.0075 |
| Myelin-associated oligodendrocytic basic protein | Mobp | 39.6 | 0.0111 |
| Insulin-like growth factor 2 mRNA binding protein 1 | Igf2bp1 | 39.3 | 0.0147 |
| Tripartite motif-containing 71 | Trim71 | 38.9 | 0.0119 |
| NK-3 transcription factor, locus 1 (Drosophila) | Nkx3-1 | 38.4 | 0.0468 |
| Myelin transcription factor 1-like | Myt1l | 37.7 | 0.0156 |
| Apolipoprotein B | Apob | 37.6 | 0.0193 |
| S100 calcium binding protein A9 (calgranulin B) | S100a9 | 36.8 | 0.0081 |

**Supplementary Table 3** Top 20 known genes downregulated in dental pulp cell cultures of *Mmp13*^-/-^ mice. Normalization of three independent biological replicates in each group was carried out using the DESEQ method with a threshold of one. Genes that were differentially expressed (>2.0 absolute fold change, >1 log_2_) in the *Mmp13^-/-^* relative to WT control were identified after passing a t-test (p<0.05), post-hoc multiple test (Storey with Bootstrapping) with a corrected q value of 0.05.

| **Gene** | **Gene name** | **Fold change** | **q value** |
| --- | --- | --- | --- |
| Tripartite motif-containing 12A | Trim12a | -43.9 | 0.0076 |
| Trophoblast glycoprotein-like | Tpbgl | -10.8 | 0.0054 |
| MicroRNA 6236 | Mir6236 | -8.9 | 0.0126 |
| nuclear encoded tRNA glutamic acid 4 (anticodon TTC) | n-TEttc4 | -6.7 | 0.0076 |
| Ly6/neurotoxin 1 | Lynx1 | -6.2 | 0.0048 |
| N-acetyltransferase 8-like | Nat8l | -6.1 | 0.0092 |
| MicroRNA 6538 | Mir6538 | -5.6 | 0.0127 |
| Sodium channel, voltage-gated, type I, beta | Scn1b | -5.4 | 0.0106 |
| Transcription factor 15 | Tcf15 | -5.3 | 0.0109 |
| Leucyl-tRNA synthetase, mitochondrial | Lars2 | -5.3 | 0.0108 |
| Transmembrane protein 28 | Tmem28 | -5.3 | 0.0396 |
| Kelch-like 30 | Klhl30 | -5.2 | 0.0111 |
| Smoothelin-like 2 | Smtnl2 | -5.2 | 0.0206 |
| Hemochromatosis type 2 (juvenile) | Hfe2 | -5.2 | 0.0278 |
| Dystrophia myotonica-protein kinase | Dmpk | -5.2 | 0.0056 |
| SH3-binding domain kinase family, member 2 | Sbk2 | -5.1 | 0.0145 |
| Proline rich membrane anchor 1 | Prima1 | -5.0 | 0.0295 |
| Sema domain, transmembrane domain (TM), and cytoplasmic domain, (semaphorin) 6C | Sema6c | -5.0 | 0.0171 |
| Four and a half LIM domains 3 | Fhl3 | -5.0 | 0.0135 |
| Junctophilin 2 | Jph2 | -4.9 | 0.0141 |

**Supplementary Table 4** Over-represented upregulated pathways (genes > 2-fold) in *Mmp13^-/-^* mice dental pulp cells, analysed using PathVisio software (version 3.3.0+). In order to remove falsely over-represented pathways results were filtered by the number genes changed (>2), z-score (>1.96), and permuted p-values (<0.05). These pruned results minimize redundant terms and pathways.

| **Pathway** | **Number changed** | **% changed** | **z-score** | **P value (permuted)** |
| --- | --- | --- | --- | --- |
| GPCRs, Class A Rhodopsin-like | 225 | 65 | 12.91 | <0.0001 |
| Odorant GPCRs | 188 | 66 | 11.93 | <0.0001 |
| GPCRs, Other | 156 | 68 | 11.55 | <0.0001 |
| Non-odorant GPCRs | 266 | 54 | 10.66 | <0.0001 |
| Peptide GPCRs | 70 | 74 | 8.87 | <0.0001 |
| Blood Clotting Cascade | 20 | 80 | 5.3 | <0.0001 |
| Glucocorticoid and Mineralcorticoid Metabolism | 13 | 85 | 4.64 | <0.0001 |
| Complement and Coagulation Cascades | 61 | 52 | 4.43 | <0.0001 |
| Monoamine GPCRs | 33 | 61 | 4.3 | <0.0001 |
| DNA Replication | 41 | 51 | 3.45 | 0.001 |
| Biogenic Amine Synthesis | 15 | 60 | 2.84 | 0.006 |
| Dopaminergic Neurogenesis | 30 | 50 | 2.8 | 0.004 |
| Steroid Biosynthesis | 13 | 62 | 2.77 | 0.009 |
| Cytokines and Inflammatory Response | 27 | 48 | 2.43 | 0.012 |
| SIDS Susceptibility Pathways | 61 | 41 | 2.41 | 0.013 |
| Serotonin and anxiety | 17 | 53 | 2.37 | 0.015 |
| Macrophage markers | 10 | 60 | 2.32 | 0.015 |
| Serotonin and anxiety-related events | 13 | 54 | 2.15 | 0.029 |
| Osteoclast | 14 | 50 | 1.97 | 0.031 |

**Supplementary Table 5** Top 20 over-represented down-regulated pathways (genes > 2-fold) in *Mmp13^-/-^* mice dental pulp cells, analysed using PathVisio software (version 3.3.0). In order to remove falsely over-represented pathways results were filtered by the number genes changed (>2), z-score (>1.96), and permuted p-values (<0.05). These pruned results minimize redundant terms and pathways.

| **Pathway** | **Number changed** | **% changed** | **z-score** | **P value (permuted)** |
| --- | --- | --- | --- | --- |
| Insulin Signalling | 158 | 92 | 5.64 | <0.0001 |
| EGFR1 Signalling Pathway | 175 | 91 | 5.48 | <0.0001 |
| TNF-alpha NF-kB Signaling Pathway | 184 | 90 | 5.26 | <0.0001 |
| Focal Adhesion | 185 | 87 | 4.45 | <0.0001 |
| IL-6 signalling Pathway | 99 | 92 | 4.33 | <0.0001 |
| IL-3 signalling Pathway | 100 | 91 | 4.15 | <0.0001 |
| PluriNetWork | 290 | 83 | 4.09 | <0.0001 |
| Delta-Notch Signalling Pathway | 84 | 92 | 3.93 | <0.0001 |
| Alpha6-Beta4 Integrin Signaling Pathway | 66 | 94 | 3.9 | <0.0001 |
| Focal Adhesion-PI3K-Akt-mTOR-signaling pathway | 323 | 82 | 3.76 | <0.0001 |
| Adipogenesis genes | 133 | 86 | 3.61 | 0.001 |
| Striated Muscle Contraction | 45 | 96 | 3.46 | <0.0001 |
| ESC Pluripotency Pathways | 117 | 86 | 3.35 | <0.0001 |
| Wnt signalling Pathway and Pluripotency | 97 | 88 | 3.33 | <0.0001 |
| MAPK signalling pathway | 159 | 84 | 3.33 | 0.003 |
| Integrin-mediated Cell Adhesion | 100 | 87 | 3.24 | <0.0001 |
| ErbB signaling pathway | 46 | 93 | 3.18 | <0.0001 |
| Wnt Signaling Pathway NetPath | 108 | 86 | 3.16 | 0.002 |
| MicroRNAs in Cardiomyocyte Hypertrophy | 83 | 88 | 3.15 | 0.004 |
| Dysregulated miRNA Targeting in Insulin/PI3K-AKT Signaling | 26 | 100 | 3.13 | 0.005 |
